# GlycoRNA-rich, neutrophil membrane-coated, siMT1-loaded nanoparticles mitigate abdominal aortic aneurysm progression by inhibiting the formation of neutrophil extracellular traps

**DOI:** 10.1016/j.mtbio.2025.101630

**Published:** 2025-03-04

**Authors:** Zhiwei Zhang, Tianyu Ling, Qingwei Ding, Feng Zhu, Xiaoyuan Cheng, Xiaoting Li, Teng Ma, Qingyou Meng

**Affiliations:** a Department of Vascular Surgery, General Surgery Clinical Center, Shanghai General Hospital, Shanghai JiaoTong University School of Medicine, Shanghai, China; b Translational Medical Center for Stem Cell Therapy and Institute for Regenerative Medicine, Shanghai East Hospital, Tongji University School of Medicine, Shanghai, China; c Department of Geriatrics, Shanghai General Hospital, Shanghai JiaoTong University School of Medicine, Shanghai, China

**Keywords:** Abdominal aortic aneurysm, Metallothionein 1, Neutrophil extracellular traps, GlycoRNA, Nanoparticles

## Abstract

Abdominal aortic aneurysm (AAA) is a life-threatening vascular condition. Currently, there are no clinically available pharmacological interventions that can stop the progression of AAA, primarily due to the incomplete understanding of its pathogenesis and the absence of effective drug delivery systems. The present study aimed to develop a targeted therapy for AAA through a nanomedicine approach involving site-specific regulation of neutrophil extracellular trap (NET)-related pathological vascular remodeling. We found that metallothionein 1 (MT1) was upregulated in AAA lesions in both humans and mice. MT1 also facilitated the formation of NETs and subsequently induced phenotypic transformation and apoptosis in vascular smooth muscle cells. Additional *in vivo* studies revealed that the glycoRNA-rich membranes coated siMT1-loaded poly(lactic-co-glycolic acid) (PLGA)-polyethylene glycol (PEG) nanoparticles (GlycoRNA-NP-siMT1) effectively delivered siMT1 to AAA lesions, thereby inhibiting abdominal aortic dilation. Mechanistically, GlycoRNA-NP-siMT1 mitigated pathological remodeling of the abdominal aorta by reducing neutrophil infiltration and inhibiting the formation of NETs. Our study indicates that MT1 facilitates the progression of AAA by modulating the formation of NETs. Furthermore, GlycoRNA-NP-siMT1 show an inhibitory effect on AAA progression through a dual mechanism: they competitively inhibit neutrophil infiltration and release siMT1, which subsequently suppresses NET formation.

## Graphical abstract

**Figure:**
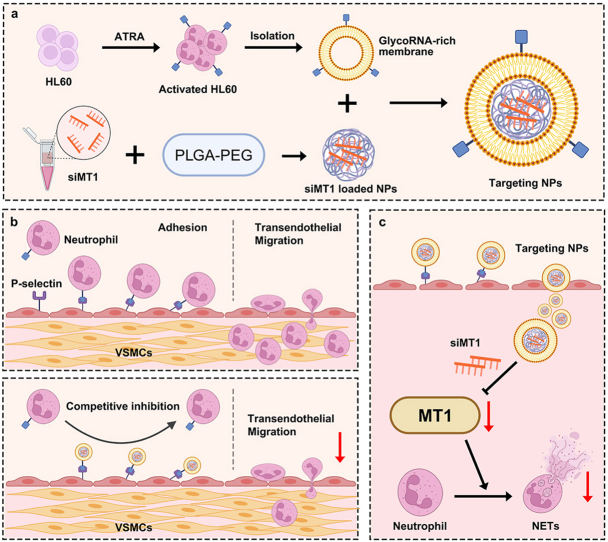

## 1 Introduction

Abdominal aortic aneurysm (AAA) is a critical vascular disorder marked by the permanent enlargement of the abdominal aorta, and it results in approximately 200,000 deaths worldwide annually [1]. Currently, open surgical repair and endovascular aortic repair are the only clinically effective treatments for large AAAs, which are defined as those with a diameter of ≥55 mm [2]. However, these procedures are invasive and are associated with a range of postoperative complications, including cardiopulmonary events, acute kidney injury, and infections. Clinical studies have indicated that surgical intervention does not provide benefits for patients with small AAAs, which are defined as those with 30–55 mm diameter, particularly when these aneurysms are still growing [3]. Hence, there is an urgent need to develop pharmacological interventions that can reverse the pathophysiology and progression of AAA.

Inflammatory cell infiltration and the subsequent local inflammatory response are the critical factors in the initiation and progression of AAA [4]. However, the specific types of immune cells and the molecular mechanisms involved in AAA formation are unclear. Recent studies have highlighted the remarkable role of neutrophils in the pathological processes associated with AAA [5]. Neutrophils are the first inflammatory cells to infiltrate the aortic wall in response to AAA risk factors; they release various proinflammatory mediators and matrix metalloproteinases (MMPs), which contribute to the apoptosis of vascular smooth muscle cells (VSMCs) and degradation of extracellular matrix [5]. Neutrophils can also generate extracellular reticular structures consisting of histones, granular proteins, and depolymerized genomic DNA through a distinct mechanism known as NETosis [6]. This process culminates in the formation of neutrophil extracellular traps (NETs). Recent evidence indicates that excessive production of NETs may facilitate vascular pathological remodeling by accelerating the death of endothelial cells (ECs) and VSMCs [5]. Additionally, some studies have reported that NETs significantly promote AAA formation, which was mitigated by administering NETosis inhibitors in murine models [7]. Nevertheless, the causes, functions, and specific molecular mechanisms underlying abnormal NET aggregation during AAA progression remain poorly characterized.

In the context of AAA therapy, it is imperative that the inhibition of NETosis is modulated in a site-specific and precise manner, given the critical role of NETs in host defense and immunomodulatory functions. In recent decades, extensive research has demonstrated that advanced nanotechnology-based nanoparticles (NPs) are a promising strategy for targeted treatment and facilitate the efficient delivery of therapeutic agents, including drugs and genetic materials, to lesion sites [8,9]. NPs can traverse damaged endothelial barriers or can be transported by inflammatory cells through a hitchhiking effect, thereby enabling targeted intervention of vascular lesions [10]. In addition to passive targeting strategies, NPs can be modified with specific molecules or cellular membranes to facilitate active targeting [11]. This approach enhances selective accumulation and improves therapeutic efficacy in various animal models of both acute and chronic vascular diseases [11,12]. Numerous efforts have been made to develop targeting ligands specifically for AAA; however, current modification strategies for NPs have not yielded satisfactory therapeutic outcomes for AAA because of limitations such as poor *in vivo* stability, low targeting efficiency, and inadequate specificity [13]. Recent studies have identified glycan-modified RNAs (glycoRNAs) located on the outer surface of mammalian cells [14]. Further investigations have indicated that glycoRNAs may play a crucial role in mediating the recruitment of immune cells to inflammation sites [15]. Thus, based on these significant findings, the modification of NP surfaces with glycoRNA-rich cell membranes could be a novel strategy to enhance the targeting efficiency of NPs for treating AAA.

Given the pathological role of NETs in the progression of AAAs and considering the therapeutic advantages offered by nanotherapies, the present study describes a nanomedicine approach for the targeted therapy of AAAs through the site-specific regulation of NET-related pathological vascular remodeling. Our findings indicate that metallothionein 1 (MT1) is upregulated in aortic aneurysm lesions and exhibits a positive correlation with NETs. We developed glycoRNA-rich membranes derived from HL60 cells to engineer siMT1-loaded poly(lactic-co-glycolic acid) (PLGA)-polyethylene glycol (PEG) nanoparticles (GlycoRNA-NP-siMT1), which function as an active targeting nanotherapy. A substantial accumulation of GlycoRNA-NP-siMT1 was observed in aneurysm tissues, and these NPs effectively mitigated AAA progression. Subsequently, we investigated the mechanisms underlying the *in vivo* efficacy of GlycoRNA-NP-siMT1, with a particular emphasis on their anti-NET effects. Mechanistically, GlycoRNA-NP-siMT1 reduced local neutrophil infiltration in AAAs through competitive inhibition and simultaneously inhibited the NETosis process through the release of loaded siMT1, thereby decreasing the local excessive accumulation of NETs in AAAs. These findings contribute to the understanding of the mechanisms involved in AAA formation and offer a novel approach for NP-targeted modification.

## 2 Materials and methods

### 2.1 Human tissue samples

The present research was conducted in compliance with the ethical standards established by the Declaration of Helsinki. Prior to study inclusion, all participants provided informed consent, and the research protocol was approved by the Ethics Committee of Shanghai General Hospital, affiliated to the Shanghai Jiao Tong University School of Medicine (Shanghai, China; Approval No.: 2023SQ189). Human AAA tissues, adjacent non-AAA tissues, and blood samples were collected from eight patients who underwent open surgical procedures for AAA between January 2021 and December 2023. The aortic tissues were stored at −8 °C or fixed with 4 % paraformaldehyde (PFA) for subsequent real-time quantitative reverse transcription polymerase chain reaction (RT-qPCR) analysis and immunofluorescence staining.

### 2.2 Bioinformatics analysis

Normal and dilated abdominal aortic tissues (n = 3 for each group) obtained from the PPE-induced mouse AAA model were prepared for RNA sequencing (RNA-seq). The RNA-seq experiments were performed by Lianchuan Biotech Co., Ltd. (Hangzhou, China) in accordance with established protocols. Differentially expressed genes (DEGs) were identified using criteria: fold change >5 and p < 0.05., employing the OmicStudio platform (https://www.omicstudio.cn/).

### 2.3 Cell culture and treatment

Human umbilical vein endothelial cells (HUVECs) and HL60 cells were obtained from the American Type Culture Collection. VSMCs and neutrophils were isolated from C57BL/6 mice. The cells were cultured in RPMI 1640 medium (Thermo Fisher Scientific, China) supplemented with 10 % fetal bovine serum. To induce NETosis, 10 μg/mL lipopolysaccharide (LPS; Absin, China) were added to the cell culture medium at specified time points. In order to silence endogenous MT1, HL60 cells were transfected with siMT1 using Lipofectamine RNAiMAX (Thermo Fisher Scientific, China) 48 h prior to the induction of NETs. To assess the internalization of NPs and conduct Transwell assay for neutrophil migration, HUVECs and VSMCs were pretreated with 50 ng/mL tumor necrosis factor-alpha (TNF-α) for 6 h at 37 °C.

### 2.4 Synthesis and characterization of siMT1-Loaded targeting NPs

*Preparation of siMT1-Loaded NPs:* PLGA-encapsulated siMT1 NPs were prepared by Xi'an Qiyue Biology (China). Initially, 20 μM siMT1 (Ribobio, China) was added dropwise to 800 μL of dichloromethane, which contained 25 mg of PLGA-PEG-MAL (MCE, USA), in a TE buffer (pH 8.0). The resulting mixture was emulsified using sonication (50 W for 1 min) to form a water-in-oil (W1/O) emulsion. Subsequently, the emulsion was diluted with 5 mL of 1 % polyvinyl alcohol (PVA 1500, w/v) and stirred with a magnetic stirring bar for 3 h at room temperature to facilitate the evaporation of dichloromethane. Finally, the PLGA NPs were collected via ultracentrifugation at 15,000 g for 15 min at 4 °C, followed by washing with deionized water and freeze-drying for 24 h. Additionally, fluorescent-labeled NPs were produced using sulfo-cyanine5 (Cy5)-labeled siMT1 or siNC.

*Preparation of glycoRNA-rich membranes:* HL60 cells were differentiated into neutrophil-like cells by using a previously described method with some modifications [16]. Various concentrations (2–10 μM) of all-trans retinoic acid (ATRA; MCE, USA) were added to the HL60 differentiation medium, and glycoRNA present on the cell surface was detected using a fluorescent probe. Both HL60 cells and glycoRNA-rich neutrophil-like cells were harvested and lysed with Tris-HCl buffer (pH 7.4) containing 0.2 mM EDTA (Absin) and a phosphatase inhibitor cocktail (Absin) under constant agitation on a shaker at 4 °C overnight. The cell lysate was sonicated on ice three times by using an ultrasonic probe at 20 % amplitude for 20 s each time. The resulting supernatant was centrifuged at 10,000×*g* for 30 min, followed by an additional round of centrifugation at 70,000×*g* for 90 min at 4 °C. Finally, the pellet was resuspended in Dulbecco's phosphate-buffered saline.

*Preparation of cell membrane-coated NPs:* Cell membranes were combined with siMT1-loaded NPs at the weight ratio of 3:1 (w/w) and subsequently subjected to 20 cycles of extrusion through nucleopore membranes with pore sizes of 400 nm initially and 200 nm later by using an Avanti mini extruder (Avanti Polar Lipids, USA). Dynamic light scattering (DLS, Malvern, UK) and transmission electron microscopy (TEM, JEOL Ltd., Japan) were utilized to assess the hydrated size, surface zeta potential, and morphology of the resulting NPs.

### 2.5 Animals

Animal experiments conducted in the present study received approval from the Animal Ethics Committee of Shanghai General Hospital, affiliated to Shanghai Jiao Tong University School of Medicine. The angiotensin II (Ang II)-induced murine AAA model was established as previously described. Briefly, male ApoE^−/−^ mice aged 10–12 weeks were anesthetized using isoflurane (RWD Life Science, China). Then, mini osmotic pumps loaded with 1000 ng min^−1^ kg^−1^ of Ang II or saline were implanted subcutaneously at the back of the neck for 28 days. An external porcine pancreatic elastase (PPE)-induced mouse AAA model was established in accordance with previously described methodologies [17]. Briefly, male mice aged 10–12 weeks were anesthetized using isoflurane (RWD Life Science, China). The infrarenal abdominal aortas were isolated and wrapped with a gelatin sponge presoaked in 10 μL PPE (7.5 mg/mL; Sigma Aldrich) for 15 min. For treating AAA, equal volumes (5 mg in 200 μL PBS) of siMT1-loaded PLGA-PEG NPs (NP-siMT1), glycoRNA-rich membrane-coated siMT1 control-loaded NPs (GlycoRNA--NP-scramble), and glycoRNA-rich membrane-coated siMT1-loaded NPs (GlycoRNA-NP-siMT1) were administered to mice through the tail vein on days 1, 3 and 7, after PPE treatment. A sham group of mice was subjected to all procedures, except for PPE treatment. The induction of the mouse AAA model utilizing CaCl_2_ was conducted in a manner analogous to that employed for induction with PPE, utilizing a CaCl_2_ concentration of 0.5 mol/L. At 28 days post-surgery, mice were euthanized humanely through isoflurane inhalation overdose (5 %), followed by cervical dislocation. Subsequently, the suprarenal abdominal aortas were harvested for further analysis. For *in vivo* localization analysis, a range of NPs loaded with Cy5 (MCE, USA) were administered to mice through the tail vein on the third day following PPE treatment. After 6 h, the abdominal aortas were excised and subsequently examined with an image visualization and infrared spectroscopy (IVIS) system (PerkinElmer, Inc., USA) together with immunofluorescence staining. An independent evaluation of toxicity associated with NPs was performed in both normal and AAA mouse models.

### 2.6 Immunofluorescence staining

Cell or tissue sections were first fixed with 4 % PFA and then permeabilized with 0.25 % Triton X-100. Subsequently, the sections were blocked with an immunostaining blocking solution. The sections were then incubated overnight at 4 °C with primary antibodies, followed by incubation for 1 h with the appropriate secondary fluorescence antibodies (Proteintech Group, China). Dihydroethidium (DHE) staining and terminal deoxynucleotidyl transferase dUTP nick end labeling (TUNEL) staining were performed using DHE and TUNEL apoptosis kits (Servicebio, China), respectively, in accordance with the manufacturer's instructions. Imaging was performed using a confocal laser scanning microscope (Leica Biosystems, Germany).

### 2.7 Histological analysis

The abdominal aortas were fixed with 4 % PFA, embedded in paraffin, and sectioned for further analysis. The serial sections were deparaffinized, rehydrated, and stained using hematoxylin and eosin (H&E) and Verhoeff-van Gieson (EVG) staining kits (all sourced from Servicebio, China) in accordance with the manufacturer's protocols. Images were captured using a Pannoramic MIDI scanner (3DHISTECH Ltd., Hungary).

### 2.8 Determination of intracellular reactive oxygen species levels

The Reactive Oxygen Species Assay Kit (Solarbio, China) was used to measure cytoplasmic reactive oxygen species (cytoROS) levels. Briefly, treated neutrophils were plated in a 24-well culture plate precoated with poly-L-lysine. Next, 200 μL of 2ʹ,7ʹ-dichlorodihydrofluorescein diacetate (DCFH-DA) solution (10 mM) was added to the culture plate, and the plate was incubated at 37 °C for 30 min. The fluorescence intensity of DCFH-DA was evaluated with a microscope to quantify the intracellular ROS levels.

### 2.9 ELISA

ELISA was conducted using an ELISA kit (Cayman, USA) in accordance with the manufacturer's instructions. Briefly, lysates from the abdominal aorta were obtained by centrifugation. The concentrations of TNF-α, interleukin-1β (IL-1β), interleukin-6 (IL-6), monocyte chemoattractant protein-1 (MCP-1) were quantified.

### 2.10 Neutrophil migration assay

HUVECs were cultured in a Transwell chamber containing 6.5 mm polycarbonate inserts with a pore size of 5.0 μm (Corning, USA) and were pretreated with TNF-α as reported previously. Equal volumes of NPs were incubated with HUVECs for 4 h. Subsequently, the culture medium in the upper insert was removed, and 2 × 10^5^ purified neutrophils suspended in 0.5 mL Hank's balanced salt solution (HBSS; Solarbio, China) were introduced into the upper insert. Additionally, 0.5 mL of HBSS supplemented with 2 mM N-formyl-Met-Leu-Phe (Selleck, USA) was added to the lower chamber. After a migration period of 2 h, the cells in the lower chamber were collected and counted.

### 2.11 Neutrophil adhesion assay

HUVECs were cultured in a 4-well chamber slides (Genebrick, China). GlycoRNA-NP-siMT1 (100 μg/mL) were incubated with HUVECs for 4 h. Subsequently, NPs was removed, 2 × 10^5^ Cy5 labeled neutrophils suspended in 1 mL HBSS were introduced into the slides. Following a 30-min co-culture, the suspended neutrophils were removed by washing and the number of adhered cells was visualized by a microscope.

### 2.12 RT-qPCR

Total RNA was extracted from cells or abdominal aortas by using TRIzol reagent (Thermo Fisher Scientific). RT-qPCR was conducted with the BeyoFast™ SYBR Green One-Step qRT-PCR Kit (Beyotime, China) on a Real-Time PCR Detection System (Bio-Rad, USA) in accordance with the manufacturer's instructions. Glyceraldehyde-3-phosphate dehydrogenase (GAPDH) was used as an internal control. The primers used in this study are detailed in Table S1.

### 2.13 Western blot

Protein isolation, quantification, and electrophoresis were conducted in accordance with previously established protocols. Glyceraldehyde-3-phosphate dehydrogenase was utilized as a loading control. The analysis of protein blots was carried out using Image Pro Plus version 6.0.

### 2.14 Statistical analysis

All quantified data are presented as mean ± standard deviation (SD). Data analysis and image generation were conducted using GraphPad Prism 9.0 (GraphPad Software, USA). Unpaired Student's t-test was used for quantitative comparisons between two groups. One-way ANOVA or two-way ANOVA followed by a post-hoc Bonferroni test was used for comparisons among multiple groups. Kaplan-Meier analysis was used for the survival analysis. A p-value of <0.05 was considered statistically significant.

## 3 Results

### 3.1 MT1 is upregulated and is associated with the formation of NETs in both human and murine AAA tissues

**Fig. 1:**
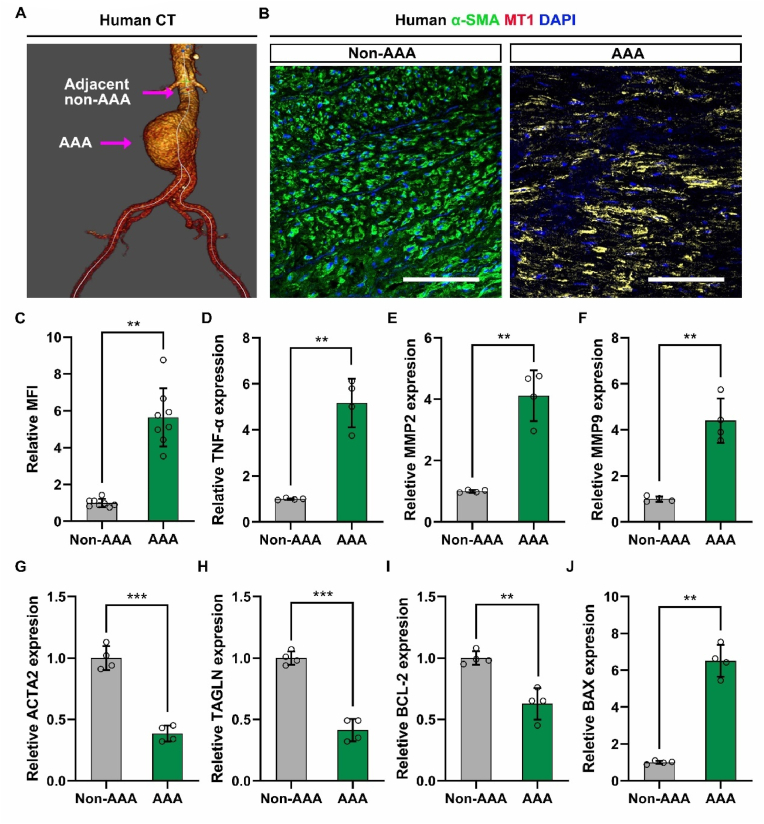
**MT1 expression in human AAA tissues. (A)** Three-dimensional reconstructed CT image of AAA. **(B)** Human AAAs were subjected to immunofluorescence (IF) staining for detecting MT1 (red) and α-SMA (green, VSMC marker) expression, and the nuclei were counterstained with DAPI. Scale bar = 100 μm. **(C)** Quantification of MT1 mean fluorescence intensity (MFI) (n = 8). **(D**–**J)** The mRNA expression levels of TNF-α, MMP2, MMP9, ACTA2, TAGLN, BCL-2, and BAX were assessed by RT-qPCR (n = 4). Data are expressed as mean ± SD. P-values were calculated by unpaired Student's t-test. ∗∗P < 0.01 and ∗∗∗P < 0.001. MT1 = Metallothionein 1; AAA = abdominal aortic aneurysm; α-SMA = α-smooth muscle actin; VSMCs = vascular smooth muscle cells; DAPI = 4′,6-Diamidino-2′-phenylindole; MFI = mean fluorescent intensity; TNF-α = tumor necrosis factor-α; MMP2/9 = matrix metallopeptidase 2/9; ACTA2 = Actin Alpha 2; TAGLN = transgelin; BCL-2 = B-cell lymphoma-2; BAX = BCL-2 associated X protein. (For interpretation of the references to colour in this figure legend, the reader is referred to the Web version of this article.)

RNA-seq was conducted to identify candidate genes potentially associated with AAA. DEGs between AAA samples and normal controls were identified by the bioinformatics analysis. A total of 337 DEGs were identified and the *MT1* gene expression was significantly upregulated in AAA samples (Figs. S1A and B). Previous research has established that MT1 plays a significant role in the regulation of inflammation, which may influence the progression of AAA. Consequently, we selected MT1 as a candidate gene for investigation in this study. Further RT-qPCR experiments confirmed the increased expression of MT1 in different AAA mouse models (Figs. S1C–E). Subsequently, we investigated the expression levels of AAA-related genes, including *TNF-α*, matrix metallopeptidase 2/9 (*MMP2/9*), actin Alpha 2 (*ACTA2*), transgelin (*TAGLN*), B-cell lymphoma-2 (*BCL-2*), and BCL-2 associated X protein (*BAX*) in human samples. Fig. 1A shows a three-dimensional reconstructed computed tomography (CT) image of the abdominal aortas from the patients from whom the AAA tissues were derived. Immunofluorescence staining showed that the fluorescence intensity of MT1 was significantly elevated in AAA tissues as compared to that in adjacent non-AAA tissues (Fig. 1B and C). RT-qPCR analysis confirmed that the expression levels of genes related to vascular pathological remodeling in the human samples were consistent with previous reports (Fig. 1D–J). Recent studies have shown the involvement of NETs in inflammation-related diseases. Therefore, we first confirmed the presence of NETs in AAA tissues by immunofluorescence staining (Figs. S2A and B). Similar results were noted for elastase-induced mouse AAA when compared with the control group (Fig. S3).

### 3.2 Silencing of MT1 expression inhibits NETs formation

**Fig. 2:**
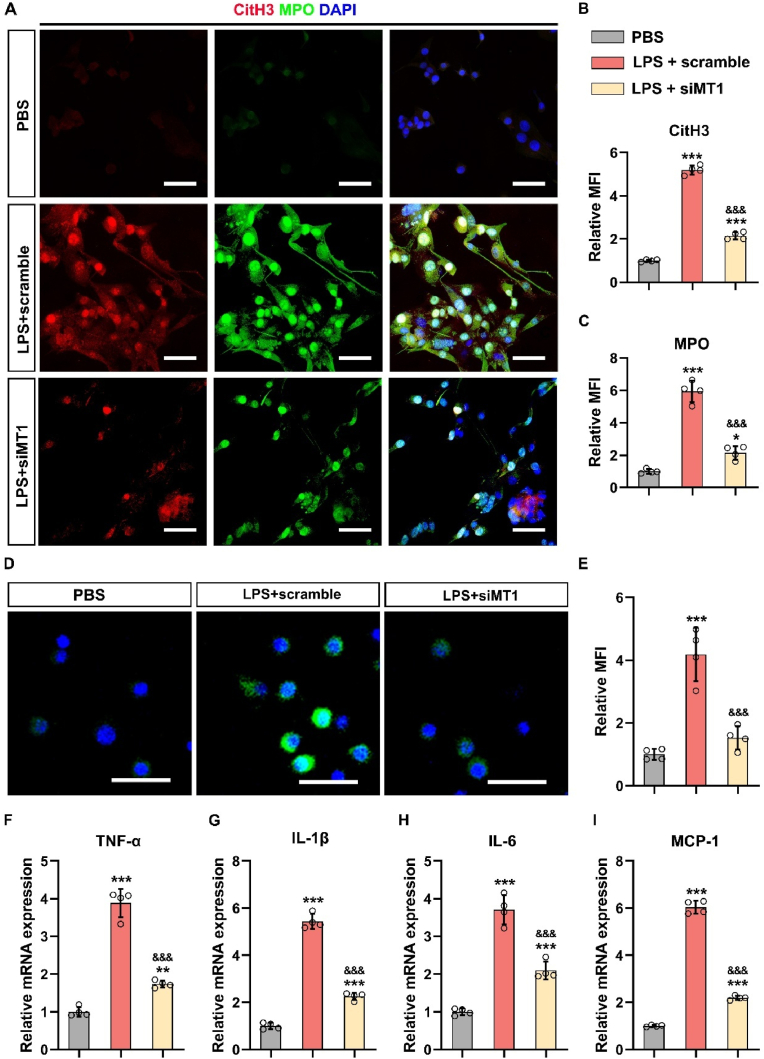
**MT1 silencing inhibits the formation of NETs. (A)** Treated neutrophils were subjected to IF staining for CitH3 (red) and MPO (green, both are NET markers) expression at 8 h, and the nuclei were counterstained with DAPI. Scale bar = 50 μm. **(B and C)** Quantification of Quantification of the fluorescence intensity (n = 4). **(D)** Treated neutrophils were stained with cytoROS probes (green, scale bar = 50 μm). **(E)** Quantification of cytoROS MFI (n = 4). **(F**–**I)** Determination of the levels of inflammatory factors, including TNF-α, IL-1β, IL-6, and MCP-1, by ELISA. Data are presented as mean ± SD. P values were determined using one-way ANOVA with the post-hoc Bonferroni test. ∗∗P < 0.01 and ∗∗∗P < 0.001. NETs = neutrophil extracellular traps; CitH3 = citrullinated histone H3; MPO = myeloperoxidase; cytoROS = cytoplasmic reactive oxygen species; ELISA = enzyme linked immunosorbent assay. (For interpretation of the references to colour in this figure legend, the reader is referred to the Web version of this article.)

The role of MT1 in promoting NETosis was assessed through loss-of-function assays. HL60 cells were subjected to treatment with either siMT1 or a scrambled control for a duration of 48 h prior to the induction of NETs. Immunofluorescence staining was conducted at 8 h post-LPS treatment. CitH3 and MPO were used as specific markers for detecting NETs. Our findings indicated that neutrophils treated with PBS did not release NETs, whereas neutrophils treated with LPS exhibited substantial NET formation. The knockdown of endogenous MT1 resulted in a decreased induction of NETs in response to LPS (Fig. 2A–C). Additionally, the effects of MT1 on the release of ROS and proinflammatory factors, including TNF-α, IL-1β, IL-6 and MCP-1 were evaluated. Following MT1 silencing administration, both ROS (Fig. 2D and E) and proinflammatory factor levels (Fig. 2F–I) were significantly decreased. Previous studies have shown MT1 is a crucial positive regulator of NF-kB activity. To elucidate the molecular pathway through which MT1 regulates the formation of NETs, we investigated the protein expression of p65 using Western blot analysis. Our findings indicated that treatment with siMT1 resulted in a reduction of p65 expression in neutrophils, which subsequently inhibited the formation of NETs (Fig. S4 A-D).

### 3.3 Synthesis and characterization of NPs targeting AAA

**Fig. 3:**
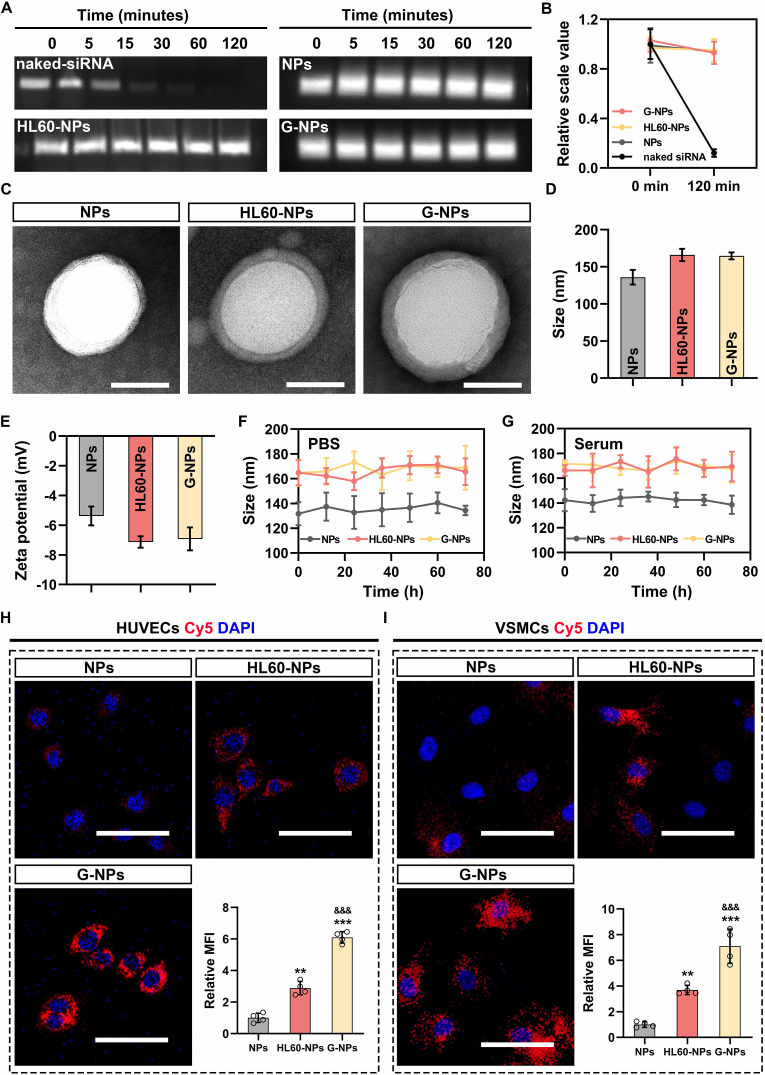
**Synthesis and characterization of GlycoRNA-NP-siMT1. (A and B)** siMT1 degradation was assessed by gel electrophoresis. **(c)** Morphology of NPs was detected by TEM. **(D and E)** Particle size and zeta potential were assessed by DLS. **(F and G)** Particle structure stability test in PBS and serum. **(H and I)** Internalization of NPs was performed in ECs and VSMCs. Data are presented as mean ± SD. P values were determined using one-way ANOVA with the post-hoc Bonferroni test. ∗∗P < 0.01 and ∗∗∗P < 0.001 *vs.* NPs; &&& P < 0.001 *vs.* HL60-NPs. HL60-NPs = HL60-NP-siMT1; G-NP = GlycoRNA-NP-siMT1; TEM = transmission electron microscopy; DLS = dynamic light scattering.

Targeted NPs were constructed using a series of systematic steps as follows: (1) preparation of siMT1-loaded PLGA-PEG NPs, (2) isolation of glycoRNA-rich cell membranes, and (3) coating of siMT1-loaded PLGA-PEG NPs with glycoRNA-rich cell membranes. Small interfering RNA (siRNA) was encapsulated within PLGA-PEG NPs as described previously. To confirm the successful loading of NPs, siMT1-loaded NPs and naked siRNA were incubated with ribonuclease (RNase). The results indicated that naked siRNA degraded rapidly, whereas the siRNA extracted from different NPs maintained structural integrity when exposed to RNase for up to 120 min (Fig. 3A and B). GlycoRNA-rich cell membranes were isolated from activated HL60 cells. As shown in Figs. S5A and B, the glycoRNA level was significantly increased following ATRA treatment and was even higher than that of primary neutrophils derived from mouse peripheral blood. The morphology of NP-siMT1, HL60-NP-siMT1, and GlycoRNA-NP-siMT1 was analyzed by transmission electron microscopy (Fig. 3C). The cell membrane coating increased the average diameter of the NPs and resulted in more negative zeta potential (Fig. 3D and E). After prolonged storage in PBS and serum, the NPs maintained a relatively constant size, indicating satisfactory stability (Fig. 3F and G). The siMT1 release profile was also determined (Fig. S6). The cellular uptake of the NPs was evaluated in HUVECs, VSMCs, and neutrophils. GlycoRNA-NP-siMT1 exhibited higher efficiency in siRNA delivery as compared to HL60-NP-siMT1, as evidenced by a stronger relative fluorescence intensity (Fig. 3H and Fig. S7A).

### 3.4 GlycoRNA-rich neutrophil-like cell membrane enhances the targeting capability of NPs *in vivo*

**Fig. 4:**
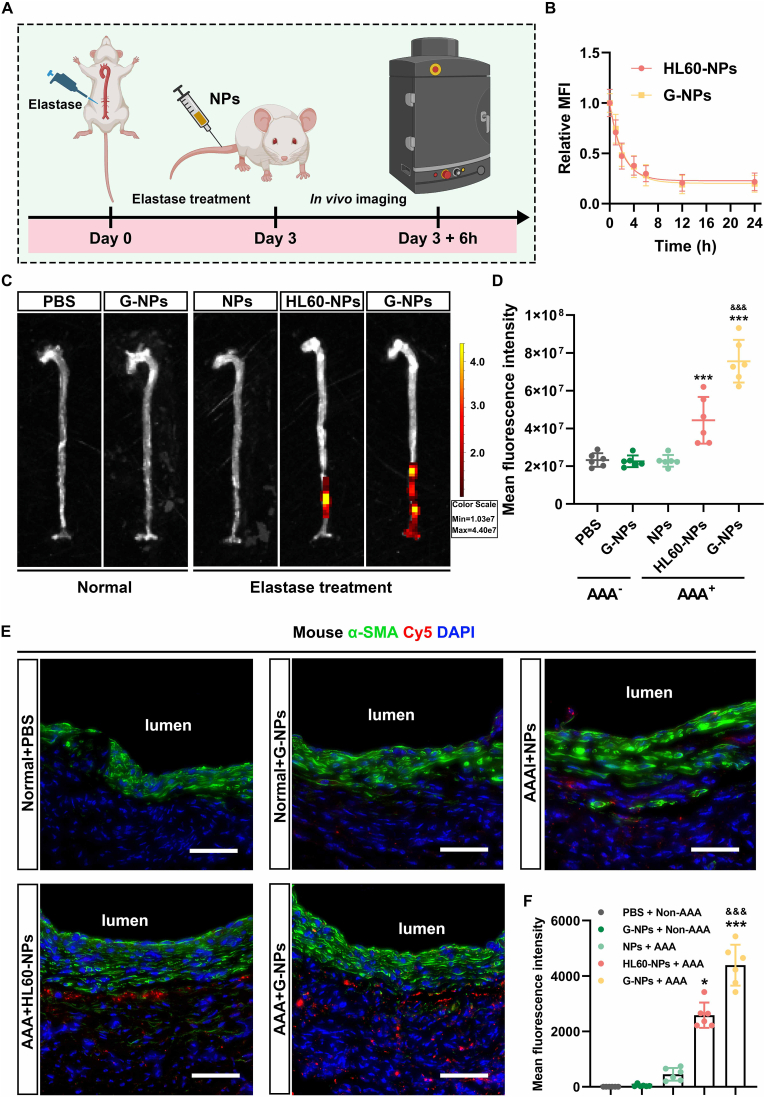
***In vivo* targeting analysis of NPs. (A)** Timeline of *in vivo* imaging. **(B)** Fluorescence degradation curve of NPs. **(C)** Detection of *in vivo* location of Cy5-labeled NPs (red) by IVIS. **(D)** Quantification of the fluorescence intensity of Cy5 (n = 6). **(E)** Detection of the location of Cy5-labeled NPs in the abdominal aorta (VSMC marker; green) by IF staining. Scale bar = 50 μm. **(F)** Quantification of the fluorescence intensity of Cy5 (n = 6). Data are presented as mean ± SD. P values were determined using one-way ANOVA with the post-hoc Bonferroni test. ∗P < 0.05, ∗∗P < 0.01 and ∗∗∗P < 0.001 *vs.* PBS. &&& P < 0.001 *vs.* HL60-NPs. (For interpretation of the references to colour in this figure legend, the reader is referred to the Web version of this article.)

The capability of NPs to target AAA was assessed using a murine AAA model (Fig. 4A). For *in vivo* localization analysis, the NPs were labeled with Cy5, and the fluorescence degradation curve of the NPs is shown in Fig. 4B. Bioluminescence analysis indicated that the GlycoRNA-NP-siMT1 did not aggregate within the non-AAA aorta. Both HL60-NP-siMT1 and GlycoRNA-NP-siMT1 were accumulated in AAA tissues; however, GlycoRNA-NP-siMT1 exhibited a higher relative radiant efficiency in these tissues as compared to HL60-NP-siMT1 (Fig. 4C and D). Additionally, the results of immunofluorescence staining revealed a higher accumulation of Cy5-labeled GlycoRNA-NP-siMT1 in AAA tissues, as evidenced by an increased fluorescence intensity (Fig. 4E and F).

### 3.5 GlycoRNA-NP-siMT1 administration attenuates AAA formation in a murine model

**Fig. 5:**
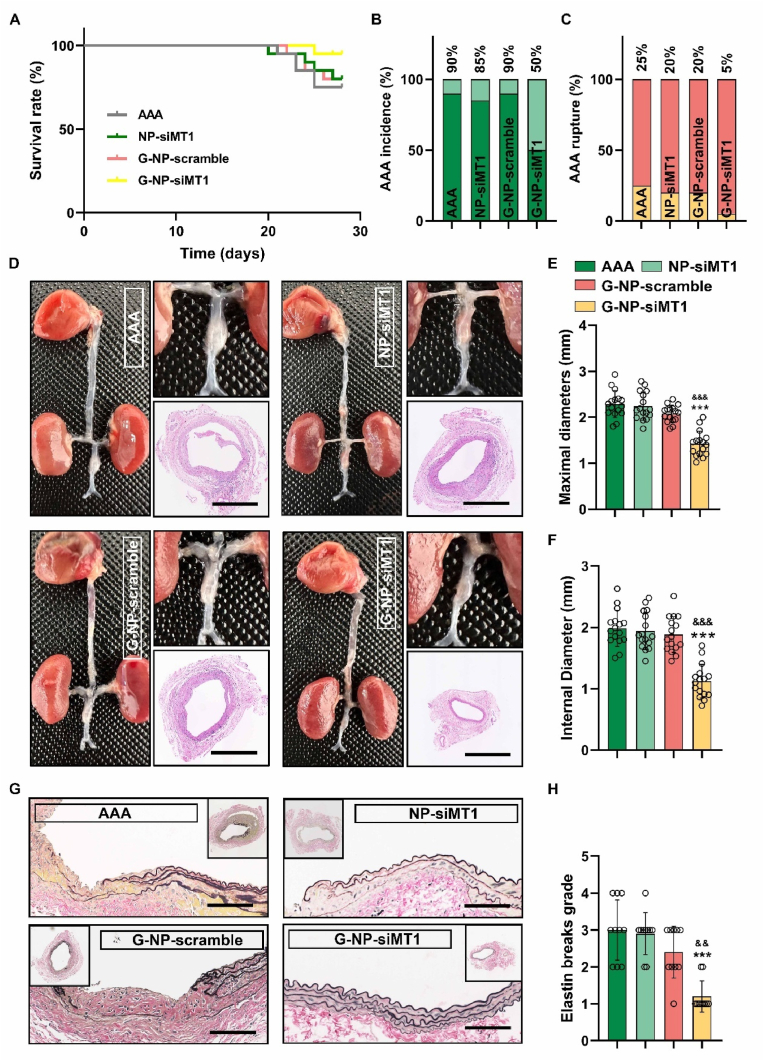
**Treatment with GlycoRNA-NP-siMT1 prevents AAA progression. (A)** Survival rate curve (n = 20). **(B)** Quantification of the aneurysm incidence in each group (n = 20). **(C)** Quantification of the aneurysm rupture in each group (n = 20). **(D)** Representative image of the sacrificed aortas. **(E and F)** Quantification of the maximum aortic diameter and inner aortic diameter**. (G)** Elastin degradation detected by EVG staining. **(H)** Quantification of the elastin degradation score. Data are presented as mean ± SD. P values were determined using Kaplan-Meier analysis or one-way ANOVA with the post-hoc Bonferroni test. ∗P < 0.05, ∗∗P < 0.01 and ∗∗∗P < 0.001 vs. PBS. && P < 0.01, &&& P < 0.001 vs. G-NP-scramble. EVG = Elastic Van Gieson.

The therapeutic efficacy of GlycoRNA-NP-siMT1 was assessed using a PPE-induced AAA mice model. Following AAA induction, the mice were assigned to four groups according to the treatment: PBS, NP-siMT1, GlycoRNA-NP-scramble, and GlycoRNA-NP-siMT1. RT-qPCR and WB analysis confirmed a reduction in MT1 expression in the AAA tissues (Figs. S8A–C). On day 28, the overall survival rate of mice in the GlycoRNA-NP-siMT1 group was significantly higher than that in the PBS, NP-siMT1, GlycoRNA-NP-scramble groups (Fig. 5A). A noteworthy finding was that GlycoRNA-NP-siMT1 administration significantly reduced the incidence of AAA and the rupture rate as compared to those of the PBS group (Fig. 5B and C). The degree of aortic dilation was evaluated by measuring the maximum diameter and internal diameters of the AAA. The abdominal aorta in the GlycoRNA-NP-siMT1 group showed the smallest maximum diameter and internal diameter (Fig. 5D–F). Furthermore, we confirmed the inhibitory effect of GlycoRNA-NP-siMT1 on abdominal aortic dilatation in the Ang II- and CaCl_2_-induced AAA model. GlycoRNA-NP-siMT1 demonstrated a significant reduction in AAA progression across all tested models (Fig. S9). Additionally, EVG staining (Fig. 5G and H), showed that GlycoRNA-NP-siMT1 mitigated the PPE-induced reduction in the fragmentation of elastic fibers.

### 3.6 GlycoRNA-NP-siMT1 administration reduces NET formation in AAA lesions

**Fig. 6:**
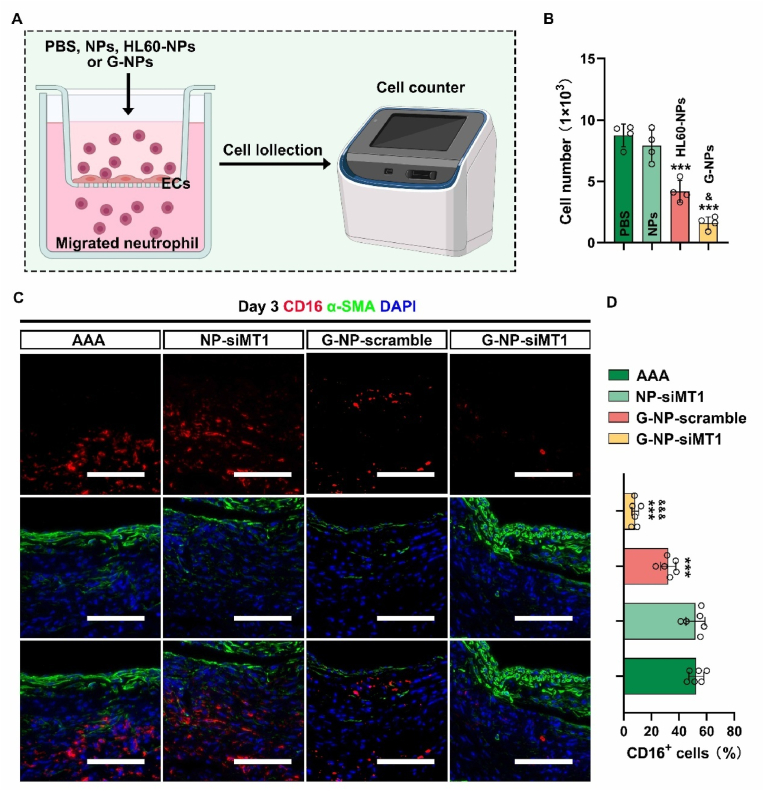
**GlycoRNA-NP-siMT1 treatment alleviates neutrophil infiltration in AAAs. (A)** Timeline of chemical treatments. **(B)** Count of migrating cells (n = 4) **(C)** AAA sections were subjected to IF staining for detecting CD16 (red, neutrophils) and α-SMA (green, VSMC marker) expression, and the nuclei were counterstained with DAPI. Scale bar = 100 μm. **(D)** Quantification of CD16 MFI (n = 6). Data are presented as mean ± SD. P values were determined using one-way ANOVA with the post-hoc Bonferroni test. ∗∗∗P < 0.001 vs. PBS. & P < 0.05, &&& P < 0.001 vs. G-NP-scramble. (For interpretation of the references to colour in this figure legend, the reader is referred to the Web version of this article.)

**Fig. 7:**
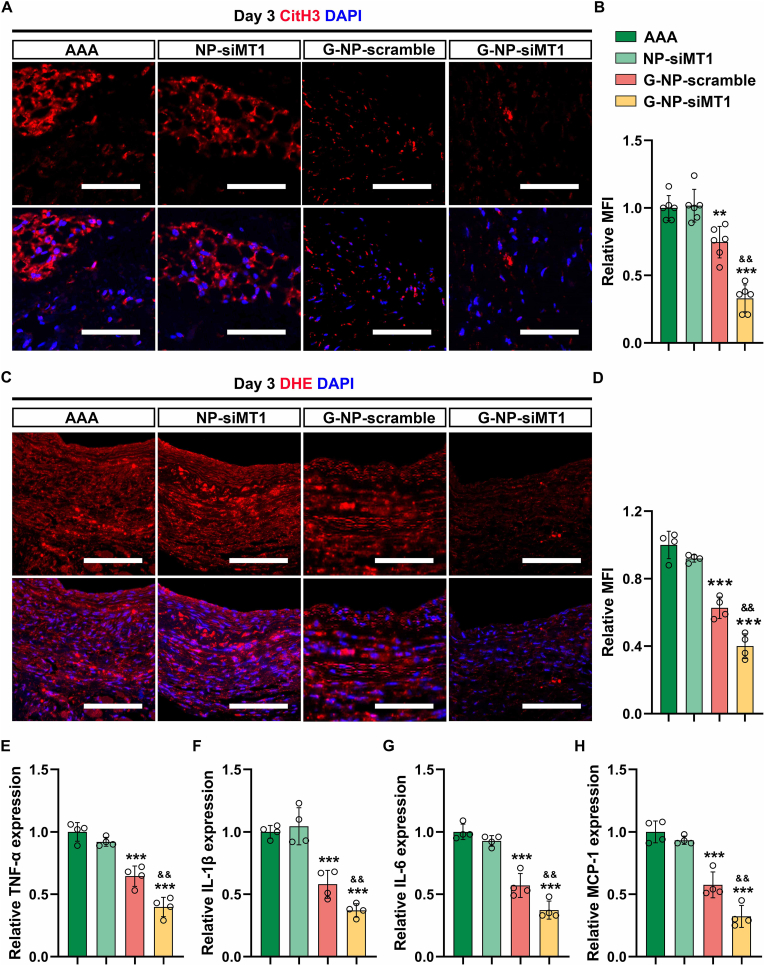
**Treatment with GlycoRNA-NP-siMT1 inhibits the formation of NETs in AAAs. (A)** AAA sections were subjected to IF staining for detecting CitH3 (red, NET marker) expression, and the nuclei were counterstained with DAPI. Scale bar = 100 μm. **(B)** Quantification of CitH3 MFI (n = 6). **(C)** AAA sections were subjected to IF staining for detecting DHE (red, ROS marker) expression, and the nuclei were counterstained with DAPI. Scale bar = 100 μm. **(D)** Quantification of DHE MFI (n = 6). **(E**–**H)** Determination of the level of inflammatory factors, including TNF-α, IL-1β, IL-6, and MCP-1, by ELISA. Data are presented as mean ± SD. P values were determined using one-way ANOVA with the post-hoc Bonferroni test. ∗∗∗P < 0.001 vs. PBS. && P < 0.01, &&& P < 0.001 vs. G-NP-scramble. DHE = Dihydroethidium. (For interpretation of the references to colour in this figure legend, the reader is referred to the Web version of this article.)

An *in vitro* neutrophil migration assay suggested that GlycoRNA-NP-siMT1 significantly reduced the number of migrated cells in an *in vitro* neutrophil migration model (Fig. 6A and B). Neutrophil adhesion assays similarly demonstrated that GlycoRNA-NP-siMT1 exert an inhibitory effect on the adhesion of neutrophils to endothelial cells (Fig. 10SA and B). In the *in vivo* assay, the regulatory role of GlycoRNA-NP-siMT1 in the formation of NETs was examined using a mouse model of AAA. Our findings indicated that both GlycoRNA-NP-scramble and GlycoRNA-NP-siMT1 significantly reduced neutrophil infiltration in AAA tissues, as evidenced by a decrease in CD16 fluorescence intensity observed on day 3 post-AAA induction (Fig. 6C and D). Additionally, by using CitH3 as a specific marker for NET detection, NET formation was assessed by immunofluorescence staining on day 3 following AAA induction. GlycoRNA-NP-scramble and GlycoRNA-NP-siMT1 treatments reduced the fluorescence intensity of CitH3. Notably, the fluorescence intensity of NET formation-associated proteins was significantly lower in the GlycoRNA-NP-siMT1 group than in the GlycoRNA-NP-scramble group (Fig. 7A and B). Furthermore, we evaluated the levels of ROS and proinflammatory factors in AAA lesions. ROS production was detected by DHE staining and was markedly inhibited by GlycoRNA-NP-siMT1 treatment as compared to treatment with PBS, NP-siMT1, and GlycoRNA-NP-scramble (Fig. 7C and D). The assessment of proinflammatory factors, including TNF-α, IL-1β, IL-6, and MCP-1, showed similar trends (Fig. 7E–H). Collectively, these results suggest that GlycoRNA-NP-siMT1 inhibits the localized overaccumulation of NETs in AAA by attenuating neutrophil infiltration and NET formation.

### 3.7 GlycoRNA-NP-siMT1 reduces phenotypic switch and apoptosis of VSMCs by regulating NETs

**Fig. 8:**
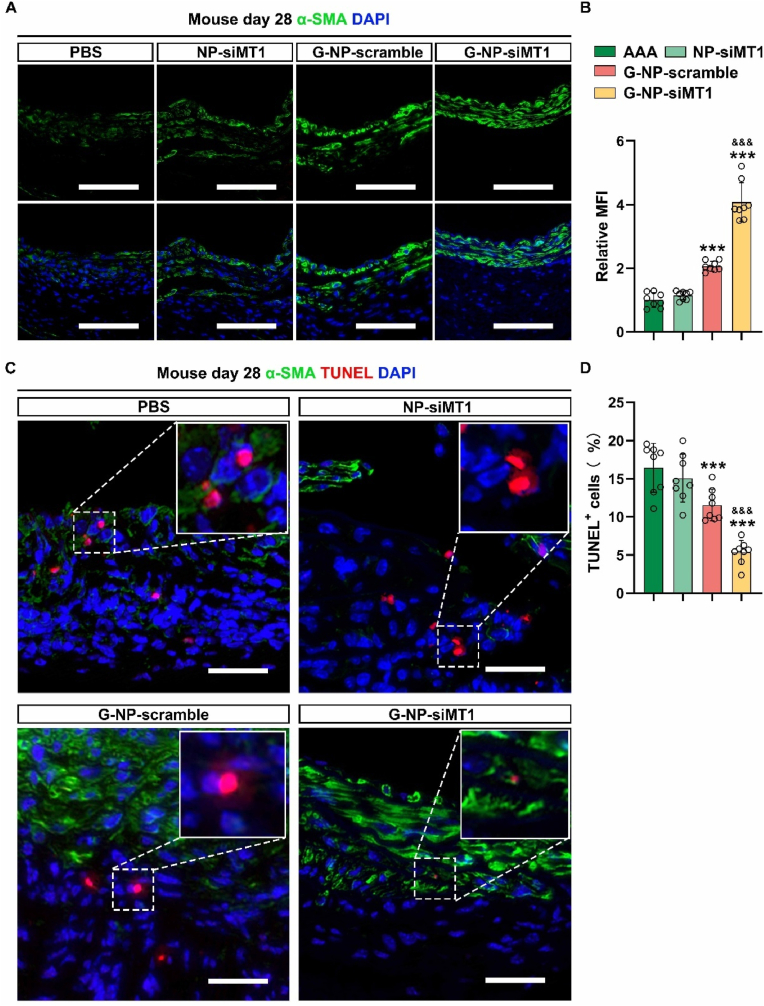
**Treatment with GlycoRNA-NP-siMT1 attenuates the phenotypic switching and apoptosis of VSMCs in AAA. (A)** AAA sections were subjected to IF staining for detecting α-SMA (green, marker for phenotypic switching of VSMCs) expression, and the nuclei were counterstained with DAPI. Scale bar = 100 μm. **(B)** Quantification of α-SMA MFI (n = 8). **(C)** AAA sections were subjected to IF staining for TUNEL (red, apoptosis marker) and α-SMA (green, VSMC marker) expression, and the nuclei were counterstained with DAPI. Scale bar = 100 μm. **(D)** Quantification of TUNEL + cells (n = 8). Data are presented as mean ± SD. P values were determined using one-way ANOVA with the post-hoc Bonferroni test. ∗∗P < 0.01, ∗∗∗P < 0.001 vs. PBS. &&& P < 0.001 vs. G-NP-scramble. (For interpretation of the references to colour in this figure legend, the reader is referred to the Web version of this article.)

Given the critical role of the phenotypic switch of VSMCs in AAA progression, we quantified the level of the contractile phenotype marker α-SMA in AAA tissues of mice on day 28 post-induction. The results of immunofluorescence staining showed a significant increase in the fluorescence intensity of α-SMA in the GlycoRNA-NP-siMT1 group as compared to that in the PSB, NP-siMT1, and G-NP-scramble groups (Fig. 8A and B). Furthermore, Western blot analysis indicated that GlycoRNA-NP-siMT1 treatment preserved the expression of α-SMA and SM22a while inhibiting the expression of MMP2, MMP9, OPN, and vimentin in the mouse AAA model (Figs. S8C–I). Previous studies have established a strong association between AAA rupture and apoptosis of VSMCs in the abdominal aorta. Therefore, we further assessed TUNEL-positive VSMCs in AAA tissues by immunofluorescence staining. As illustrated in Fig. 8C and D, the GlycoRNA-NP-siMT1 group exhibited a reduction in TUNEL-positive VSMCs, thus providing evidence for VSMC apoptosis inhibition by GlycoRNA-NP-siMT1.

**Fig. 9:**
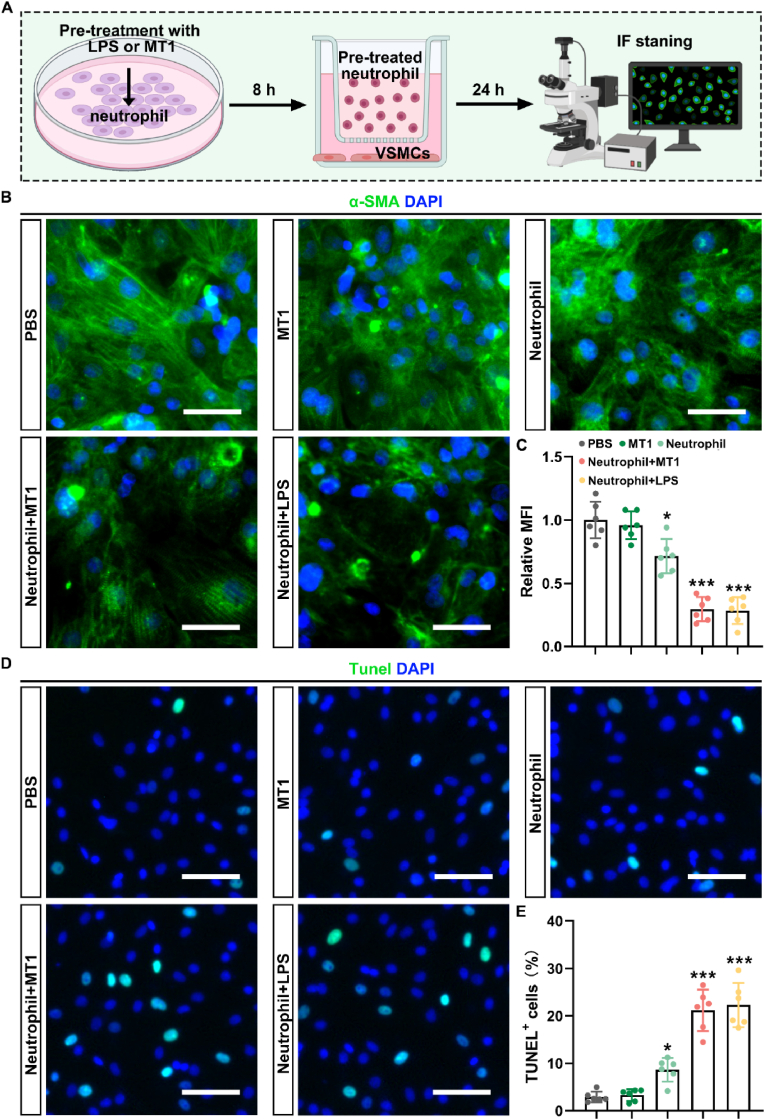
**MT1 promotes the phenotypic switching and apoptosis of VSMCs by regulating the formation of NETs. (A)** Timeline of chemical treatments. **(B)** Cells were subjected to IF staining for α-SMA (green, phenotypic switching marker for VSMCs) expression, and the nuclei were counterstained with DAPI. Scale bar = 50 μm. **(C)** Quantification of α-SMA MFI (n = 6). **(D)** Cells were subjected to IF staining for TUNEL (green, apoptosis marker) expression, and the nuclei were counterstained with DAPI. Scale bar = 50 μm. **(E)** Quantification of TUNEL^+^ cells (n = 6). Data are presented as mean ± SD. P values were determined using one-way ANOVA with the post-hoc Bonferroni test. ∗P < 0.05, ∗∗∗P < 0.001 *vs.* PBS. (For interpretation of the references to colour in this figure legend, the reader is referred to the Web version of this article.)

To further confirm that the inhibition of VSMC phenotypic switching and apoptosis by GlycoRNA-NP-siMT1 is mediated by modulating NET formation, we conducted *in vitro* gain-of-function experiments (Fig. 9A). Our findings indicate that treatment with MT1 alone did not reduce the expression of α-SMA, a synthetic phenotype marker, in VSMCs. However, co-culturing VSMCs with MT1-treated neutrophils significantly decreased α-SMA levels as compared to those in the control group (Fig. 9B and C). Similar outcomes were observed in the cell apoptosis assay (Fig. 9D and E). These results suggest that MT1 is required to mediate the production of NETs to effectively regulate phenotypic switching and apoptosis of VSMCs.

**Fig. 10:**
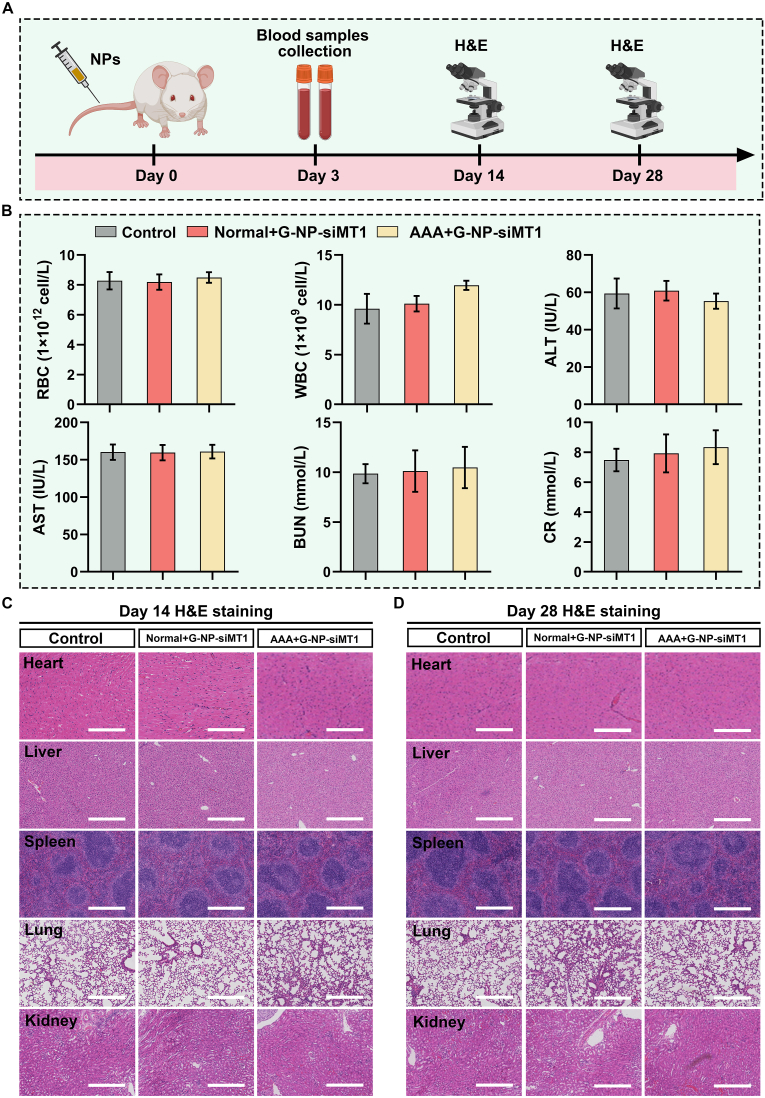
**Safety assessment of GlycoRNA-NP-siMT1 treatment. (A)** Timeline of safety assessment. **(B)** Results of blood cell analysis and blood biochemistry tests (n = 4). **(C)** H&E staining of the major organs in mice (n = 4). RBC = red blood cell; WBC = white blood cell; AST = aspartate transaminase; ALT = alanine Aminotransferase; BUN = Blood urea nitrogen; CR = creatinine. (For interpretation of the references to colour in this figure legend, the reader is referred to the Web version of this article.)

### 3.8 Safety assessment of GlycoRNA-NP-siMT1

We conducted *in vivo* safety tests of GlycoRNA-NP-siMT1 in mice. The timeline for the safety test is shown in Fig. 10A. None of the mice exhibited severe symptoms or mortality, indicating a potential absence of *in vivo* side effects. Blood samples were collected for laboratory analysis. Blood cells and the results of blood biochemistry tests showed no abnormalities (Fig. 10B). On the 14th and 28th day after NP treatment, mice were euthanized, and their major organs, including the heart, liver, spleen, lungs, and kidneys, were harvested for H&E staining. No discernible evidence of significant organ damage was observed in H&E staining (Fig. 10C and D). These findings collectively suggest that GlycoRNA-NP-siMT1 exhibit no significant *in vivo* toxicity in healthy mice at the evaluated doses, thus indicating their suitability for *in vivo* applications.

## 4 Discussion

Despite considerable efforts to manage AAA, a life-threatening condition, specific pharmacological treatments for controlling the progression of AAA pathology and reducing the risk of rupture in clinical settings remain unavailable. In the present study, we used a novel targeted molecule, glycoRNA, to modify anti-NET NPs and assessed the safety and efficacy of this GlycoRNA-NP-siMT1 therapy for AAA. The main findings of our study are as follows: (1) MT1 expression was significantly elevated and correlated with NET formation in both human and murine AAA tissues; (2) glycoRNA modification markedly enhanced the accumulation of anti-NETs NPs at AAA lesions; (3) GlycoRNA-NP-siMT1 administration attenuated aortic dilatation and reduced the incidence of rupture in a PPE-induced murine AAA model; and (4) GlycoRNA-NP-siMT1 inhibited excessive NET accumulation at AAA lesions through a dual mechanism involving the reduction of neutrophil infiltration and the inhibition of the NETosis process. These findings provide compelling evidence that GlycoRNA-NP-siMT1 may represent a promising therapeutic strategy for managing AAA.

In recent years, RNA interference-based therapeutic strategies have emerged as a promising approach for treating cardiovascular diseases (CVDs), including AAAs [[18], [19], [20]]. However, the translational potential of these therapies is limited because of the lack of effective delivery platforms that facilitate the targeted transfer of siRNA therapeutics to AAA lesions [18]. Advances in targeted nanomedicine have shown that this field has significant potential to address the abovementioned challenge [21]. Research initiatives have been undertaken to develop targeted siRNA delivery systems specifically designed for treating AAAs [22]. Nevertheless, although certain delivery systems have demonstrated some degree of cell-specific targeting *in vitro*, the majority of them have exhibited insufficient efficacy in *in vivo* AAA models. To date, there are few reports of NP-based delivery of siRNA to AAAs with high efficiency. In the present study, we constructed a PLGA-PEG NP platform and subsequently coated these NPs with glycoRNA-rich cell membranes to facilitate the efficient delivery of siRNA therapeutics to AAA lesions. To the best of our knowledge, this approach represents the first instance wherein glycoRNA has been used as a targeting moiety for modifying NPs. Our results confirmed that glycoRNA-modified NPs can be efficiently internalized by injured ECs and VSMCs *in vitro*. More importantly, glycoRNA-modified NPs can aggregate at the site of abdominal aortic injury as early as 3 days post-elastase injury, which is earlier than that achieved by previously developed AAA-targeting systems. This early aggregation is critical for the timely control of AAA progression and the inhibition of aortic dilatation and potential rupture.

Neutrophils exhibit a diverse array of functional roles, particularly through the formation of NETs, which are significantly implicated in the pathogenesis of AAAs [7,23]. A recent study reported a notable increase in NET markers in the plasma of patients diagnosed to have AAA as compared to that in healthy individuals [24]. This finding is consistent with our observations reported in the previously mentioned study, which demonstrated that the expression of NET-associated proteins was significantly elevated in the abdominal aortic tissue of patients with AAA. Although NETs serve as important indicators of AAA occurrence and prognosis, the precise mechanisms through which NETs regulate AAA formation remain unelucidated. Our findings suggest that NETs contribute to abdominal aortic dilatation by promoting the transition of VSMCs from a contractile phenotype to a synthetic phenotype. Furthermore, subsequent research has established that NETs facilitate the rupture of AAAs by inducing apoptosis in VSMCs during the terminal stages of the aneurysm. Therefore, the progression of AAA may be effectively mitigated through the regulation of local neutrophil accumulation and the suppression of NET production.

In humans, metallothioneins (MTs) are encoded by a gene family located on chromosome 16q13, which consists of at least 11 functional members [25]. The biological functions of the MT family are well documented, as these proteins exhibit a high affinity for heavy metals, thereby providing protective mechanisms for cells and tissues against the toxic effects of heavy metal exposure [25]. MTs have been implicated in various diseases, including cancers, neurological disorders, and CVDs [[26], [27], [28]]. However, their role in AAA remains ambiguous. Several studies have established that elevated expression of MT1 is associated with the promotion of inflammation-related diseases, such as atopic dermatitis and inflammatory bowel diseases. Conversely, other research suggests that MT1 may exert a protective effect against aneurysms through its antioxidative properties [29]. This apparent contradiction may be attributed to the fact that MT1 may assume distinct roles at various stages of disease progression. This study examines the role of MT1 in modulating the inflammatory microenvironment within AAA tissues. The results of our *in vitro* analysis suggest that MT1 does not directly affect the phenotypic transition of VSMCs or their cellular homeostasis. Moreover, further investigations confirmed that MT1 modulates the vascular pathological remodeling associated with AAA by influencing the formation of NETs.

The present study has some limitations. First, considering that the characteristics of elastase perfusion-induced AAA correspond with the activation of the inflammatory response and the stimulation of the protein hydrolysis cascade, we used elastase perfusion-induced mice, a widely accepted model for AAA, in our research. Hence, it is imperative that our findings should be corroborated in alternative AAA models, including those induced by angiotensin II and calcium chloride. Second, although our results substantiate the role of MT1 in promoting the formation of NETs in AAA tissues, further investigations are required to understand the precise mechanism underlying its action.

Here, we developed a glycoRNA-based NP platform that facilitates the targeted delivery of siRNA to AAAs. These NPs reduce the production of NETs by competitively inhibiting the local aggregation of neutrophils within AAAs, while concurrently releasing siRNA that targets MT1. These mechanisms further prevent the pathological remodeling of the abdominal aorta by suppressing the phenotypic transformation and apoptosis of VSMCs. The findings of the present study offer novel gene targets and delivery platforms for treating AAAs.

## CRediT authorship contribution statement

**Zhiwei Zhang:** Writing – review & editing, Writing – original draft, Software, Resources, Project administration, Investigation, Formal analysis, Data curation, Conceptualization. **Tianyu Ling:** Writing – original draft, Software, Investigation, Data curation, Conceptualization. **Qingwei Ding:** Writing – original draft, Software, Data curation, Conceptualization. **Feng Zhu:** Software, Writing – review & editing. **Xiaoyuan Cheng:** Software, Writing – review & editing. **Xiaoting Li:** Writing – original draft, Visualization, Validation, Supervision, Software, Methodology, Investigation. **Teng Ma:** Writing – original draft, Software, Project administration, Methodology, Investigation, Conceptualization. **Qingyou Meng:** Writing – review & editing, Writing – original draft, Resources, Project administration, Conceptualization.

## Declaration of competing interest

The authors declare that they have no known competing financial interests or personal relationships that could have appeared to influence the work reported in this paper.

## Data Availability

Data will be made available on request.
