# Supplementary material for: GlycoRNA-rich, neutrophil membrane-coated, siMT1-loaded nanoparticles mitigate abdominal aortic aneurysm progression by inhibiting the formation of neutrophil extracellular traps
Source: Mater Today Bio. 2025 Mar 4;31:101630. doi: 10.1016/j.mtbio.2025.101630 (PMC11929896; doi:10.1016/j.mtbio.2025.101630)
Supplement: Multimedia component 1 [file mmc1.pptx]

## Slide 1
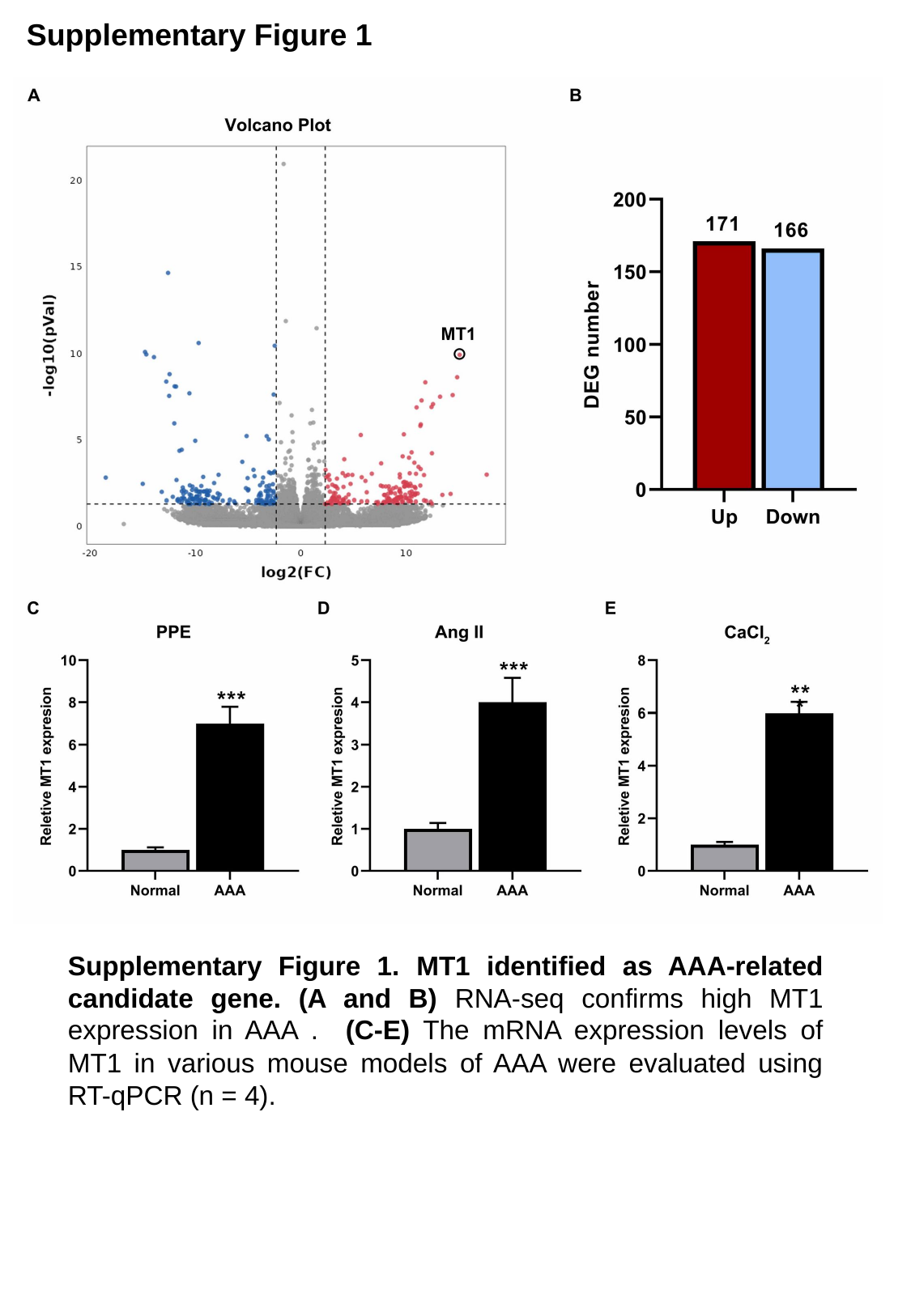

Supplementary Figure 1
Supplementary Figure 1. MT1 identified as AAA-related candidate gene. (A and B) RNA-seq confirms high MT1 expression in AAA . (C-E) The mRNA expression levels of MT1 in various mouse models of AAA were evaluated using RT-qPCR (n = 4).

## Slide 2
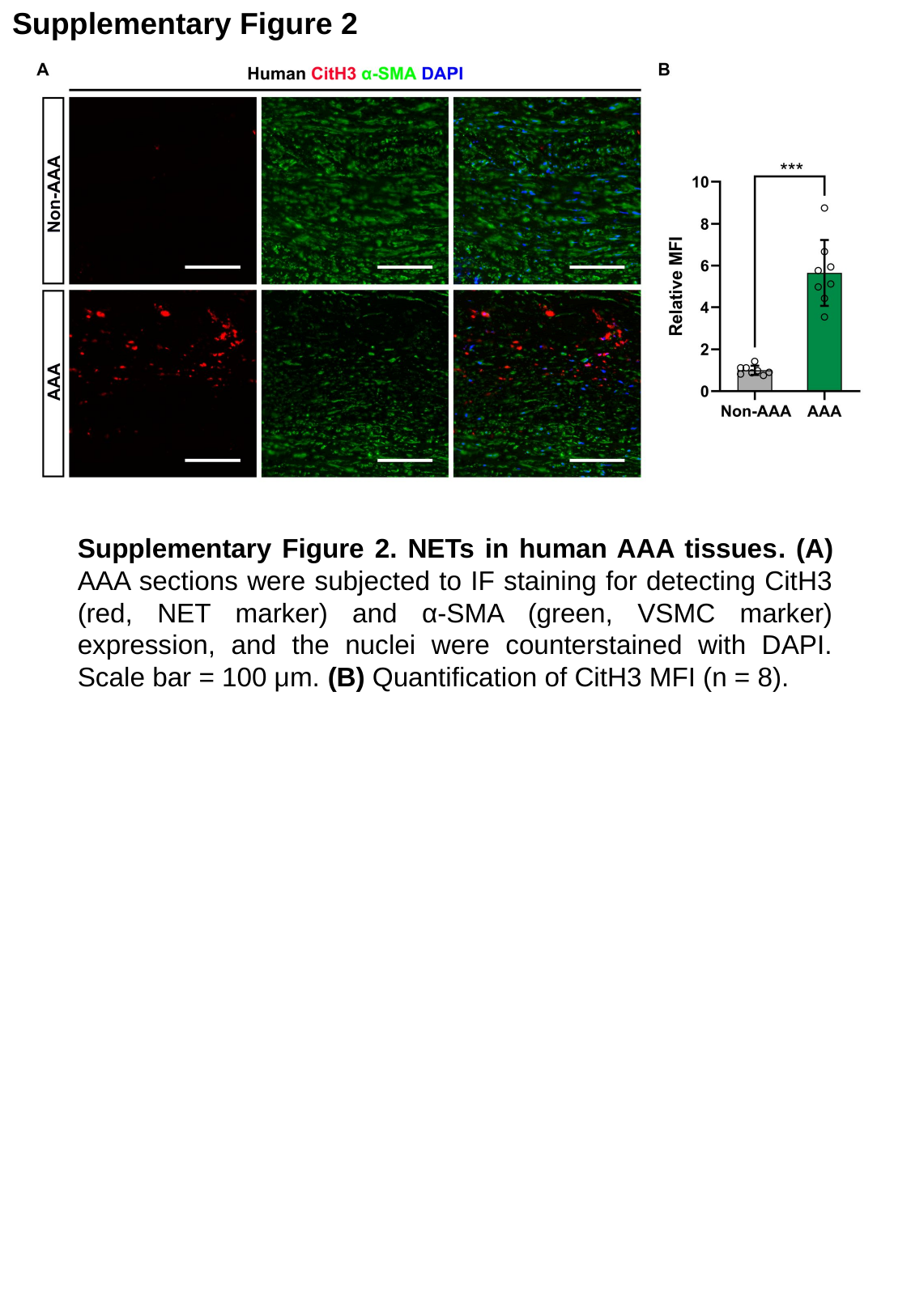

Supplementary Figure 2
Supplementary Figure 2. NETs in human AAA tissues. (A) AAA sections were subjected to IF staining for detecting CitH3 (red, NET marker) and α-SMA (green, VSMC marker) expression, and the nuclei were counterstained with DAPI. Scale bar = 100 μm. (B) Quantification of CitH3 MFI (n = 8).

## Slide 3
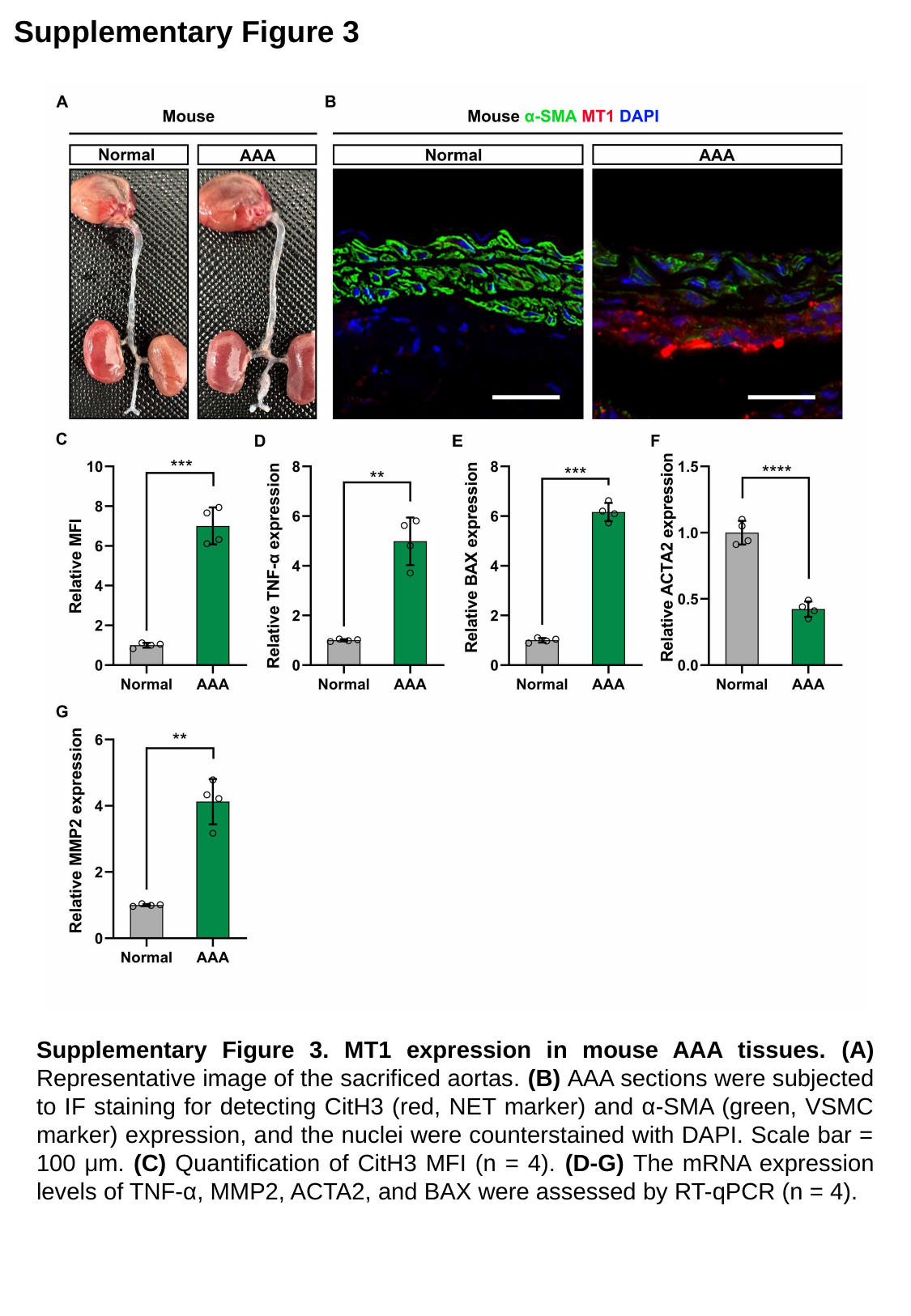

Supplementary Figure 3
Supplementary Figure 3. MT1 expression in mouse AAA tissues. (A) Representative image of the sacrificed aortas. (B) AAA sections were subjected to IF staining for detecting CitH3 (red, NET marker) and α-SMA (green, VSMC marker) expression, and the nuclei were counterstained with DAPI. Scale bar = 100 μm. (C) Quantification of CitH3 MFI (n = 4). (D-G) The mRNA expression levels of TNF-α, MMP2, ACTA2, and BAX were assessed by RT-qPCR (n = 4).

## Slide 4
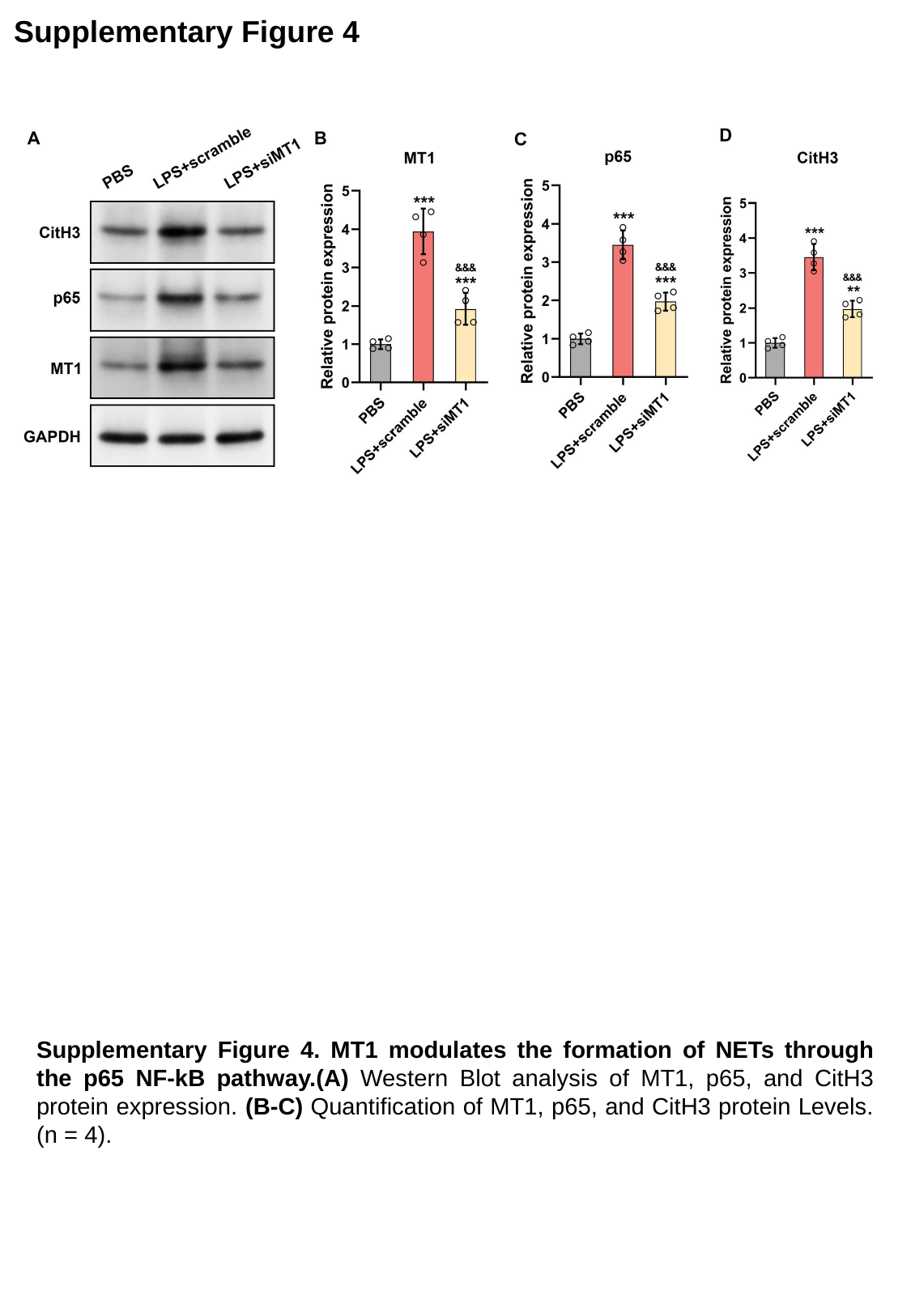

Supplementary Figure 4
Supplementary Figure 4. MT1 modulates the formation of NETs through the p65 NF-kB pathway.(A) Western Blot analysis of MT1, p65, and CitH3 protein expression. (B-C) Quantification of MT1, p65, and CitH3 protein Levels. (n = 4).

## Slide 5
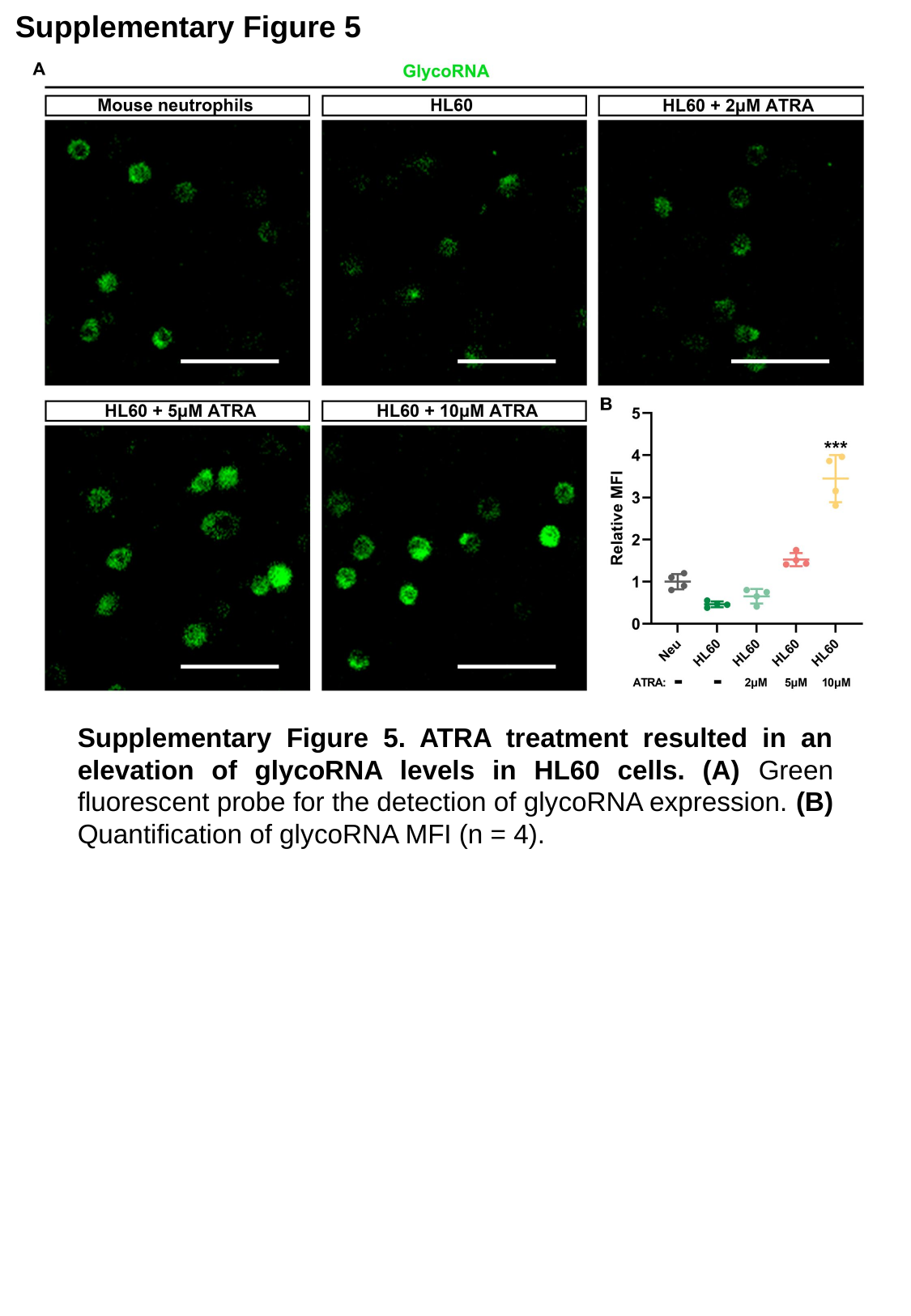

Supplementary Figure 5
Supplementary Figure 5. ATRA treatment resulted in an elevation of glycoRNA levels in HL60 cells. (A) Green fluorescent probe for the detection of glycoRNA expression. (B) Quantification of glycoRNA MFI (n = 4).

## Slide 6
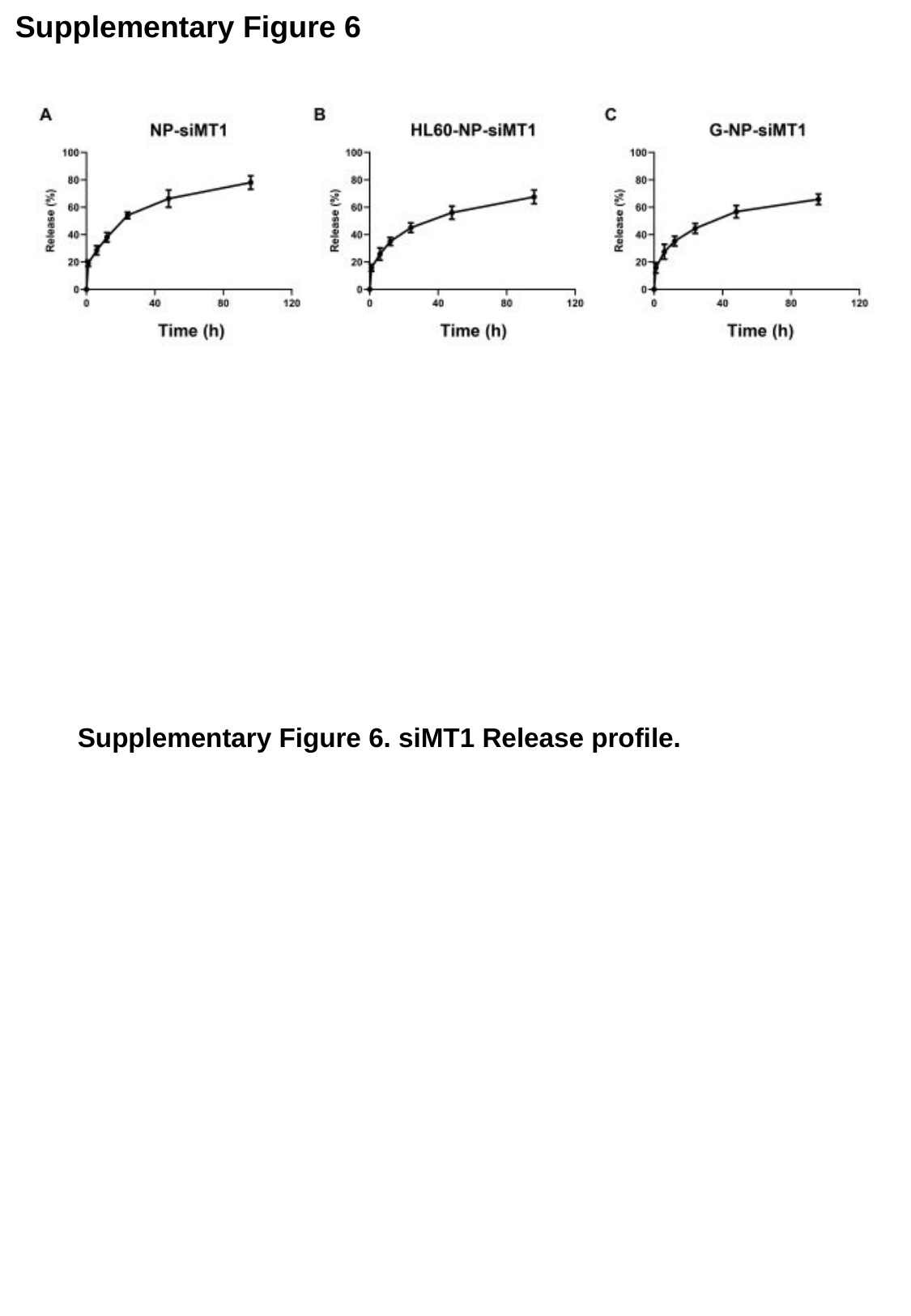

Supplementary Figure 6
Supplementary Figure 6. siMT1 Release profile.

## Slide 7
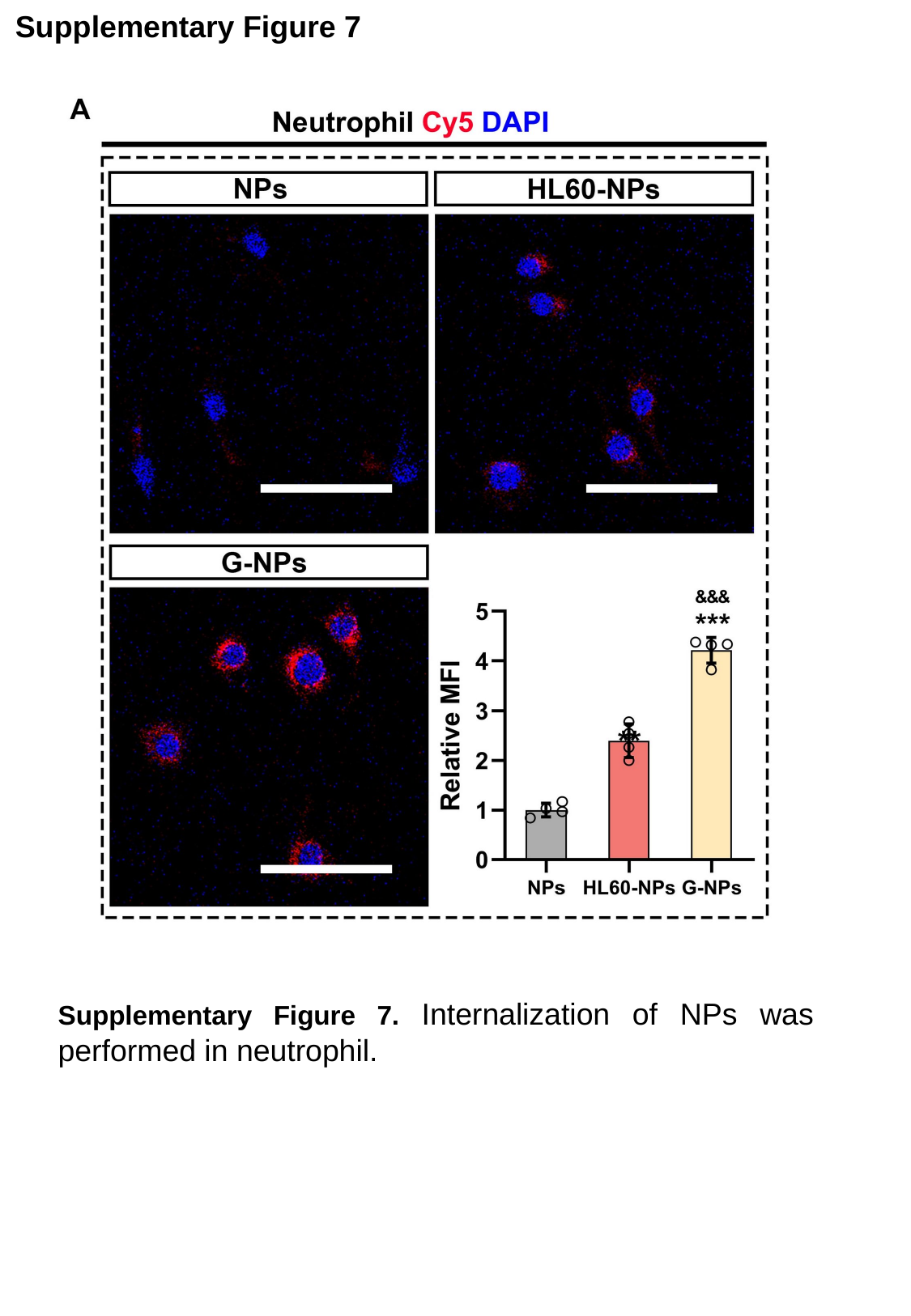

Supplementary Figure 7
Supplementary Figure 7. Internalization of NPs was performed in neutrophil.

## Slide 8
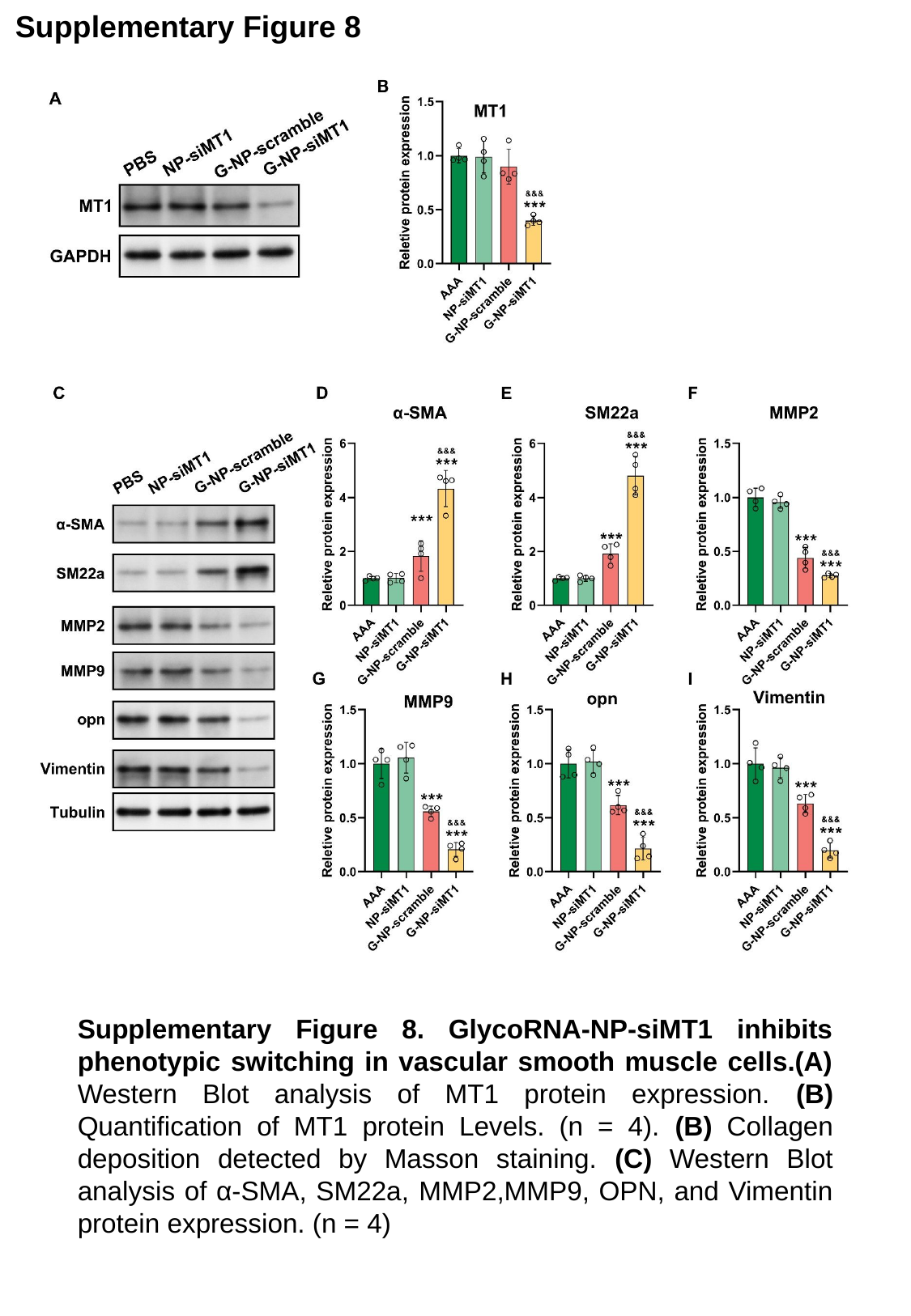

Supplementary Figure 8
Supplementary Figure 8. GlycoRNA-NP-siMT1 inhibits phenotypic switching in vascular smooth muscle cells.(A) Western Blot analysis of MT1 protein expression. (B) Quantification of MT1 protein Levels. (n = 4). (B) Collagen deposition detected by Masson staining. (C) Western Blot analysis of α-SMA, SM22a, MMP2,MMP9, OPN, and Vimentin protein expression. (n = 4)

## Slide 9
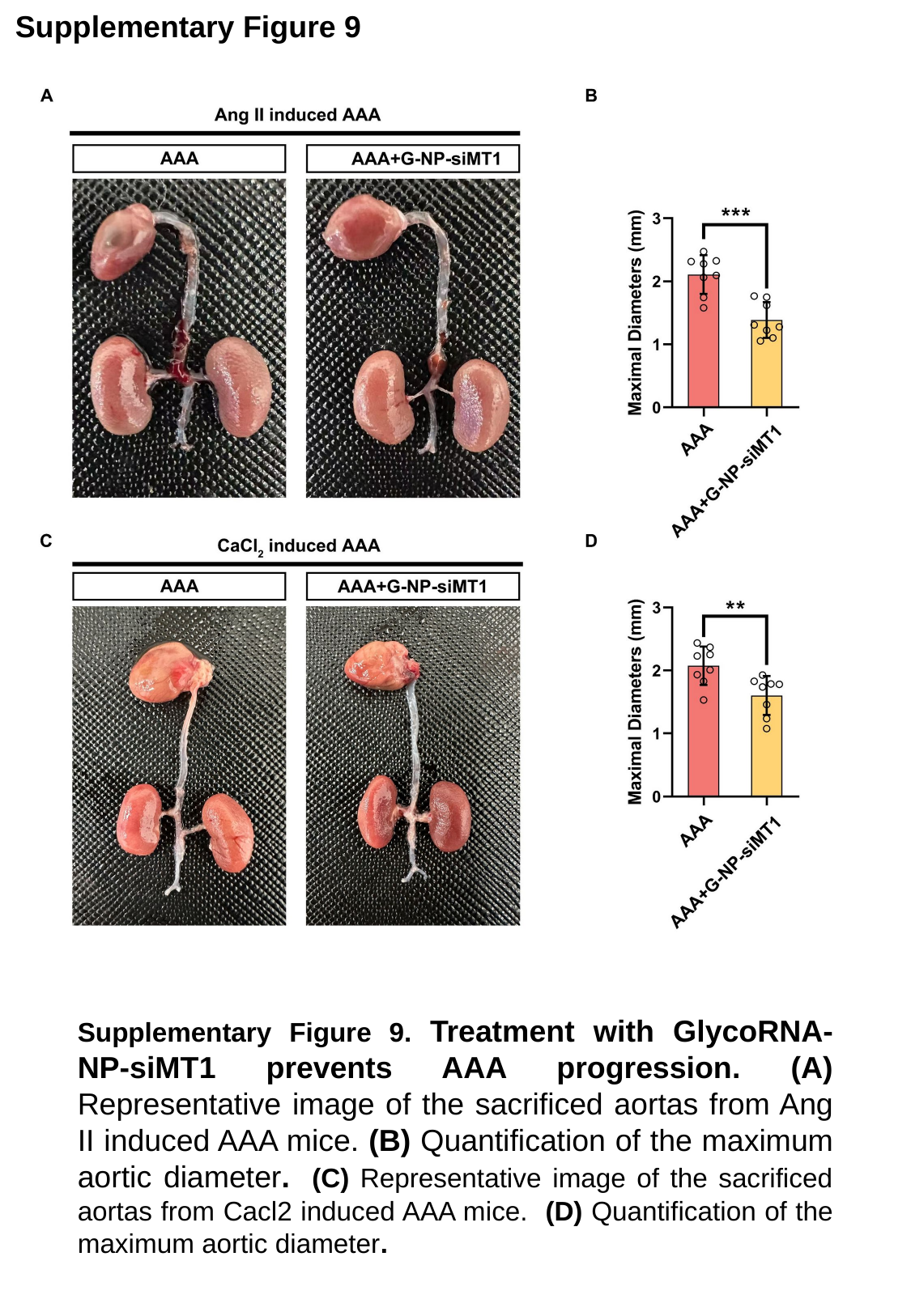

Supplementary Figure 9
Supplementary Figure 9. Treatment with GlycoRNA-NP-siMT1 prevents AAA progression. (A) Representative image of the sacrificed aortas from Ang II induced AAA mice. (B) Quantification of the maximum aortic diameter. (C) Representative image of the sacrificed aortas from Cacl2 induced AAA mice. (D) Quantification of the maximum aortic diameter.

## Slide 10
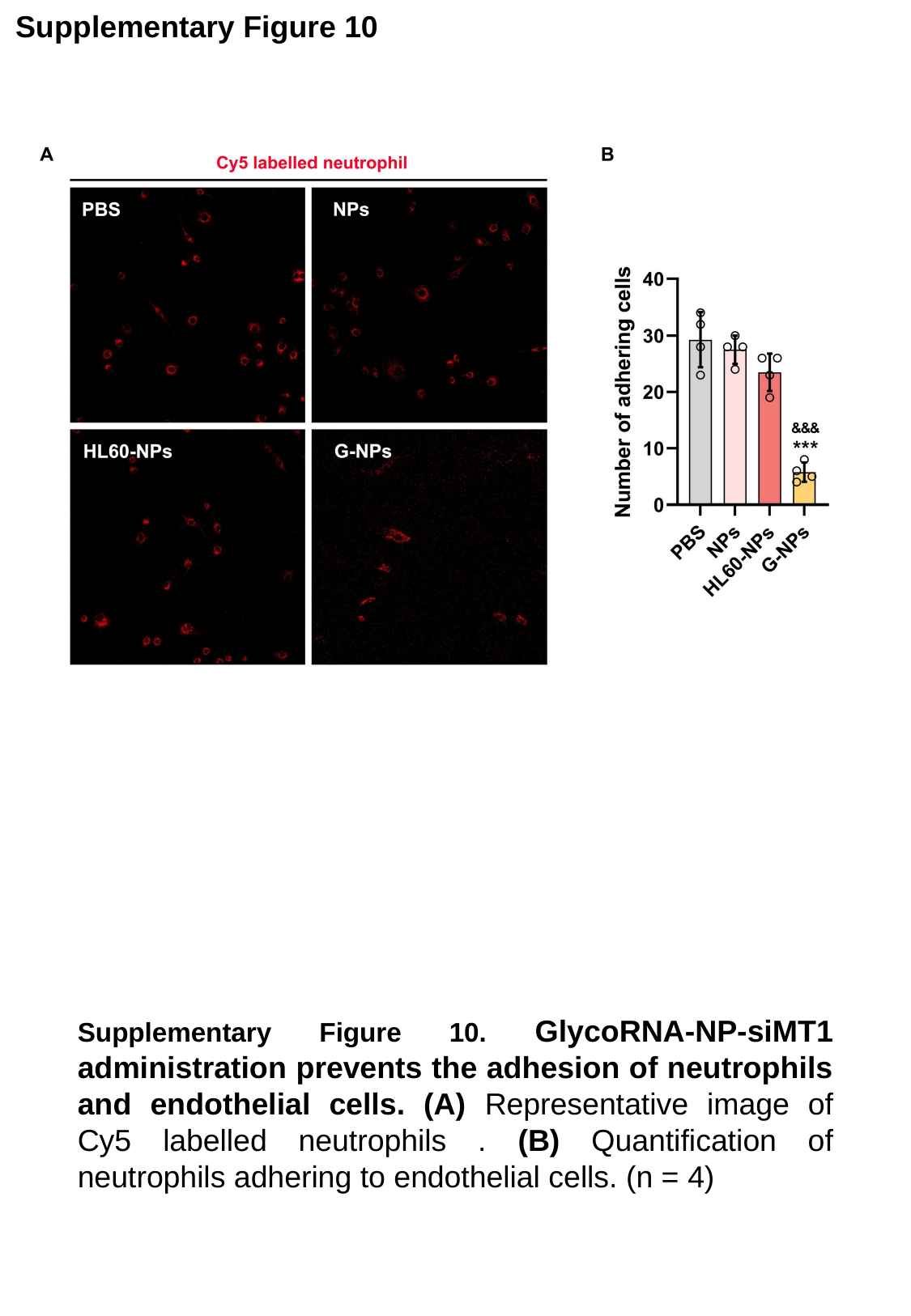

Supplementary Figure 10
Supplementary Figure 10. GlycoRNA-NP-siMT1 administration prevents the adhesion of neutrophils and endothelial cells. (A) Representative image of Cy5 labelled neutrophils . (B) Quantification of neutrophils adhering to endothelial cells. (n = 4)
