# Supplementary material for: GlycoRNA-rich, neutrophil membrane-coated, siMT1-loaded nanoparticles mitigate abdominal aortic aneurysm progression by inhibiting the formation of neutrophil extracellular traps
Source: Mater Today Bio. 2025 Mar 4;31:101630. doi: 10.1016/j.mtbio.2025.101630 (PMC11929896; doi:10.1016/j.mtbio.2025.101630)
Supplement: Multimedia component 2 [file mmc2.docx]

Supplementary Table 1. The sequences of the primers

| Gene Name | Sequence (5’- 3’) |
| --- | --- |
| *Human TNF-α* | CTCTTCTGCCTGCTGCACTTTG |
|  | ATGGGCTACAGGCTTGTCACTC |
| *Mouse TNF-α* | GGTGCCTATGTCTCAGCCTCTT |
|  | GCCATAGAACTGATGAGAGGGAG |
| *Human IL-1β* | CCACAGACCTTCCAGGAGAATG |
|  | GTGCAGTTCAGTGATCGTACAGG |
| *Mouse IL-1β* | TGGACCTTCCAGGATGAGGACA |
|  | GTTCATCTCGGAGCCTGTAGTG |
| *Human MMP2* | AGCGAGTGGATGCCGCCTTTAA |
|  | CATTCCAGGCATCTGCGATGAG |
| *Mouse MMP2* | CAAGGATGGACTCCTGGCACAT |
|  | TACTCGCCATCAGCGTTCCCAT |
| *Human MMP9* | GCCACTACTGTGCCTTTGAGTC |
|  | CCCTCAGAGAATCGCCAGTACT |
| *Mouse MMP9* | GCTGACTACGATAAGGACGGCA |
|  | TAGTGGTGCAGGCAGAGTAGGA |
| *Human ACTA2* | CTATGCCTCTGGACGCACAACT |
|  | CAGATCCAGACGCATGATGGCA |
| *Mouse ACTA2* | TGCTGACAGAGGCACCACTGAA |
|  | CAGTTGTACGTCCAGAGGCATAG |
| *Human TAGLN* | TCCAGGTCTGGCTGAAGAATGG |
|  | CTGCTCCATCTGCTTGAAGACC |
| *Mouse TAGLN* | GCAGATGGAACAGGTGGCTCAA |
|  | CCCAAAGCCATTAGAGTCCTCTG |
| *Human BAX* | TCAGGATGCGTCCACCAAGAAG |
|  | TGTGTCCACGGCGGCAATCATC |
| *Mouse BAX* | AGGATGCGTCCACCAAGAAGCT |
|  | TCCGTGTCCACGTCAGCAATCA |
| *Human BCL-2* | ATCGCCCTGTGGATGACTGAGT |
|  | GCCAGGAGAAATCAAACAGAGGC |
| *Mouse BCL-2* | CCTGTGGATGACTGAGTACCTG |
|  | AGCCAGGAGAAATCAAACAGAGG |
| *Human MT1* | AGAGTGCAAATGCACCTCCTGC |
|  | TTGTACTTGGGAGCAGGGCTGT |
| Mouse *MT1* | ACTTCAACGTCCTGAGTACC |
|  | AGCGTGTTCTGTTCGTCACAT |
