# Supplementary material for: GlycoRNA-rich, neutrophil membrane-coated, siMT1-loaded nanoparticles mitigate abdominal aortic aneurysm progression by inhibiting the formation of neutrophil extracellular traps
Source: Mater Today Bio. 2025 Mar 4;31:101630. doi: 10.1016/j.mtbio.2025.101630 (PMC11929896; doi:10.1016/j.mtbio.2025.101630)
Supplement: Multimedia component 3 [file mmc3.docx]

Supplementary Table 2

| Clinical characteristics | AAA (n=8) |
| --- | --- |
| Age(y) | 62 (54 to 71) |
| Sex |  |
| male | 4 (50%) |
| female | 4 (50%) |
| Comorbidities |  |
| Diabetes | 2 (25%) |
| Hypertension | 8 (100%) |
| Coronary heart disease | 4 (50%) |

*Data are mean or n (%).
